# Chips for Biomaterials and Biomaterials for Chips: Recent Advances at the Interface between Microfabrication and Biomaterials Research

**DOI:** 10.1002/adhm.202100371

**Published:** 2021-05-25

**Authors:** Alexander P. M. Guttenplan, Zeinab Tahmasebi Birgani, Stefan Giselbrecht, Roman K. Truckenmüller, Pamela Habibović

**Affiliations:** ^1^ Department of Instructive Biomaterials Engineering MERLN Institute for Technology‐Inspired Regenerative Medicine Maastricht University Universiteitssingel 40 Maastricht 6229ER The Netherlands

**Keywords:** biomaterials, high‐throughput screening, microfabrication, microfluidics, organ‐on‐chip

## Abstract

In recent years, the use of microfabrication techniques has allowed biomaterials studies which were originally carried out at larger length scales to be miniaturized as so‐called “on‐chip” experiments. These miniaturized experiments have a range of advantages which have led to an increase in their popularity. A range of biomaterial shapes and compositions are synthesized or manufactured on chip. Moreover, chips are developed to investigate specific aspects of interactions between biomaterials and biological systems. Finally, biomaterials are used in microfabricated devices to replicate the physiological microenvironment in studies using so‐called “organ‐on‐chip,” “tissue‐on‐chip” or “disease‐on‐chip” models, which can reduce the use of animal models with their inherent high cost and ethical issues, and due to the possible use of human cells can increase the translation of research from lab to clinic. This review gives an overview of recent developments at the interface between microfabrication and biomaterials science, and indicates potential future directions that the field may take. In particular, a trend toward increased scale and automation is apparent, allowing both industrial production of micron‐scale biomaterials and high‐throughput screening of the interaction of diverse materials libraries with cells and bioengineered tissues and organs.

## Graphical abstract

Over the last decade, the use of microfabricated chips has enabled biomaterials scientists to perform experiments at small length scales. These miniaturized experiments include the production of novel biomaterials, the screening of materials for biologically relevant properties, and the production of so‐called “organs‐on‐chip.” This review outlines progress in the field and proposes future directions for on‐chip biomaterials research.

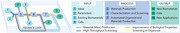

## 1 Introduction

The term “on chip” has been used in recent years to describe experiments that miniaturize studies in chemistry, biotechnology and biomedicine originally conducted at larger length scales, often using devices produced by means of microfabrication techniques adapted from the semiconductor industry.^[^ ^1^, ^2^ ^]^ These so‐called “lab‐on‐chip” systems include devices for controlled mixing and separation of chemicals, as well as a range of sensing and measurement equipment, often integrated into a single miniaturized system with external dimensions on the scale of millimeters.^[^ ^3^ ^]^ While originally developed to enable more accurate chemical analysis, they have been used for a range of applications, particularly within biochemistry and medicine.^[^ ^4^, ^5^ ^]^

In the biomedical field, these miniaturized devices include microreactors for a range of important (bio)chemical reactions,^[^ ^6^ ^]^ such as mixers to allow studies of protein dynamics over microsecond timescales,^[^ ^7^ ^]^ and devices which improve on important biochemical assays such as polymerase chain reaction (PCR)^[^ ^8^ ^]^ and enzyme‐linked immunosorbent assays (ELISA).^[^ ^9^ ^]^ As well as these biochemical methods, on‐chip tools have been used to separate mixtures of cells and other similarly sized particles in a milder way, and based on a wider range of properties, than in traditional bulk techniques such as centrifugation or magnetic‐ or fluorescence‐activated cell sorting.^[^ ^10^ ^]^ This allows single cells to be isolated and analyzed, allowing the behavior of heterogeneous populations of cells to be better understood.^[^ ^11^, ^12^ ^]^ The same microfabrication techniques used to produce chips for mixing, separation and analysis have also been used to produce on‐chip systems for cell culture. So‐called “organ‐on‐chip” environments mimic the cell culture conditions in a biological tissue or organ, which allows the reduction or replacement of animal studies.^[^ ^13^ ^]^ Alternatively, microarrays for high‐throughput studies allow large numbers of small cell samples to be interrogated in parallel in order to investigate low‐frequency events or explore a wide range of cell culture conditions.^[^ ^14^ ^]^

Microfabricated on‐chip systems are often also microfluidic systems. Microfluidics, defined as the science and technology of systems that process small (femtoliter to nanoliter) volumes of fluids in channels with dimensions of the order of tens of microns,^[^ ^15^ ^]^ has numerous advantages which make it attractive to researchers investigating the interaction between cells and their environment. Within these micron‐scale channels, fluids behave differently—flow is laminar and mixing therefore occurs only by diffusion and not by convection or turbulence.^[^ ^15^, ^16^, ^17^ ^]^ Laminar flow also allows for local control of flow rate, flow direction, and hydrodynamic pressure, as well as the production of segmented flows.^[^ ^18^, ^19^ ^]^ Fluids in these microfluidic systems behave similarly to those in certain human physiological systems, such as blood capillary flow, bone and other tissue interstitial flow, and kidney proximal tubule flow.^[^ ^20^, ^21^, ^22^, ^23^ ^]^ On‐chip systems therefore allow researchers to recapitulate the physiological microenvironment more faithfully than experiments either in conventional static cell culture dishes or in larger‐scale bioreactors in which flow is turbulent.^[^ ^24^ ^]^

The different behavior of fluids at the micron scale also allows microfluidic devices to be used to control fluid mixing and generate chemical gradients. Controlled and rapid mixing on chip enables screening of the effects of different combinations of reagents on cell behavior in an automated manner.^[^ ^25^, ^26^ ^]^ Alternatively, the ability to generate stable chemical gradients on chip has been used to identify the therapeutic window of growth factors or other soluble compounds.^[^ ^27^, ^28^, ^29^ ^]^ These gradients can also be used to recapitulate the chemical microenvironment in different (patho)physiological conditions such as a bone fracture,^[^ ^30^ ^]^ atherosclerosis^[^ ^31^ ^]^ or tumors.^[^ ^32^ ^]^

A further advantage of the different behavior of fluids in microchannels is the ability to rapidly form large numbers of stable, monodisperse droplets at a channel junction, These droplets can act as nanoliter‐scale reaction flasks or even encapsulate single cells.^[^ ^33^, ^34^ ^]^ Droplet‐based microfluidic systems for drug screening, for example, allow experiments, including those requiring cell culture over periods of more than a week, to be performed with drug and reagent consumption several orders of magnitude lower than is required for an experiment in a conventional microtiter plate.^[^ ^35^, ^36^ ^]^ In addition, these systems can be designed to enable automated and/or high‐throughput experiments to simultaneously evaluate a range of different conditions, taking advantage of the reduced volume of the experiment and the relatively compact experimental set‐up to save time and resources.^[^ ^37^ ^]^

Chips can be designed with transparent regions to allow real‐time microscopy or other imaging modalities with very low working distances,^[^ ^38^ ^]^ and can incorporate real‐time analytical tools such as miniaturized biosensors.^[^ ^39^, ^40^ ^]^ The use of real‐time, non‐invasive, and potentially automated sensing and imaging methods allows longitudinal time‐series studies of the same sample or cell population, which can give information which is unobtainable by conventional end‐point methods. Examples include real‐time electrical measurements of the cell cycle in single yeast cells,^[^ ^41^ ^]^ simultaneous measurements of cell proliferation, pH changes and oxygen consumption in fibroblast culture,^[^ ^42^ ^]^ and systems to simultaneously monitor morphology, extracellular microenvironment, and levels of biomarkers in engineered microtissues.^[^ ^43^ ^]^ In addition, miniaturization of 3‐dimensional (3D) cell culture allows for high content imaging using conventional microscopy,^[^ ^44^ ^]^ as well as non‐invasive imaging of cells cultured in a physiologically relevant microenvironment.^[^ ^45^ ^]^ Other microfluidic devices have been used to expose cells to defined mechanical cues by means of either fluid flow or integrated pneumatic or magnetic (micro)actuators in order to replicate the influence of physical forces on biological processes.^[^ ^46^, ^47^, ^48^ ^]^

Interactions between biological systems and materials, governed by phenomena occurring at small (micron‐sized) length scales similar to the size of a cell, are important in the development of biomaterials, particularly for applications such as regenerative medicine,^[^ ^49^ ^]^ drug delivery^[^ ^50^ ^]^ or gene therapy.^[^ ^51^ ^]^ On‐chip methods, which allow these phenomena to be investigated as well as enabling higher throughput, have therefore become increasingly popular for the synthesis and characterization of biomaterials and the screening of their interactions with a biological system.

On‐chip methods such as microfluidics have been exploited for the production and identification of advanced biomaterials. Microfabrication has also been used to produce and pattern surface functionalities, such as micro‐ and nanotopographies or novel surface chemistry, which are of interest in biomaterials science. As well as these studies of candidate materials and surface properties, microfabrication has been used to fabricate “chips” that mimic a biological niche in order to study corresponding cell‐material interactions in vitro. Another use of microfabrication is to produce devices made from or incorporating biomaterials for the study of cell and tissue behavior in the biomimetic microenvironment of an organ‐on‐chip. In this review article, we outline progress at this important interface in the past decade, and draw attention to areas where relatively little work has as yet been done.

## 2 Production of Biomaterials on Chip

In order to obtain materials with the right combination of chemical and physical properties for biomedical applications, capable of exerting a specific biological effect, a large parameter space needs to be explored. The mechanical properties of a candidate biomaterial are important for a variety of reasons‐ it is desirable for scaffolds used in tissue engineering to mimic the mechanical properties of the organ or tissue they replace,^[^ ^52^, ^53^ ^]^ and substrate or matrix stiffness is known to affect stem cell differentiation.^[^ ^54^, ^55^ ^]^ Materials with identical chemistry but different surface topography have been shown to have different biological effects in experiments measuring the ability of a material to induce *de novo* bone formation.^[^ ^56^, ^57^, ^58^ ^]^ Conversely, replicating the surface topography of a material while introducing different chemistry also produces a material with different biological properties from the original.^[^ ^59^, ^60^ ^]^ Therefore, experiments which compare materials that vary in one or more of these physical and chemical parameters are important to deconvolute the effect of changes in individual parameters on the biological response to the material, eventually allowing the design of biomaterials with the desired biological properties.^[^ ^61^, ^62^ ^]^

On‐chip arrays of materials enable the production of a wide variety of materials and their screening using small sample volumes. An elegant example of such an approach is the combinatorial 3D structured array platform developed by Mano and co‐workers^[^ ^63^, ^64^ ^]^ to screen biopolymer‐based nanocomposite scaffold materials for tissue regeneration applications. An alternative approach is the production of arrays of cell‐laden micro‐hydrogels having native extracellular matrix (ECM)‐like features, which has been employed by different groups to produce arrays of structurally complex microgels with different stiffness to evaluate stem cell differentiation^[^ ^65^ ^]^ and to generate large numbers of barcoded microgels mimicking specific physiological microenvironments for high‐throughput screening.^[^ ^66^ ^]^

With its increased control over mixing behavior, as well as the low sample volumes and potential for combinatorial synthesis common to all on‐chip methods, microfluidic synthesis is ideal for the exploration of the large parameter space mentioned above. In addition, micron‐scale biomaterials are advantageous for many applications. Examples include nanoparticles and microspheres for injection and micron‐scale filaments for additive manufacturing of scaffolds. Microfluidic methods lend themselves to the production of materials in these forms for use in off‐chip applications, including conventional cell culture experiments, in vivo studies in animal models, or clinical applications. In particular, biomaterials with dimensions on the micro‐ and nanoscale have been found to be of interest for a range of different applications within regenerative medicine, including as drug delivery systems^[^ ^67^ ^]^ and as building blocks for tissue engineered constructs.^[^ ^68^, ^69^ ^]^ The small size of these materials also means that they can easily be integrated into a chip either during or after synthesis to allow subsequent investigation of their properties.

### 2.1 Production of Hydrogels on Chip

Polymers, especially those that form hydrogels, are probably the class of biomaterials most commonly synthesized by on‐chip methods.^[^ ^70^, ^71^ ^]^ In particular, microfluidics is very suitable for the production of hydrogels, as the properties of diffusion‐based and therefore highly predictable mixing systems are ideal for controlled gelation. A range of gelators have been used for the production of hydrogels in microfluidic devices, including poly(vinyl alcohol) (PVA),^[^ ^72^ ^]^ alginate^[^ ^73^ ^]^ and proteins.^[^ ^74^ ^]^ Multicore droplets, sedimentation, and flow‐control have enabled researchers to produce non‐spherical micron‐sized hydrogel particles using microfluidics.^[^ ^75^, ^76^, ^77^ ^]^

**Figure 1:**
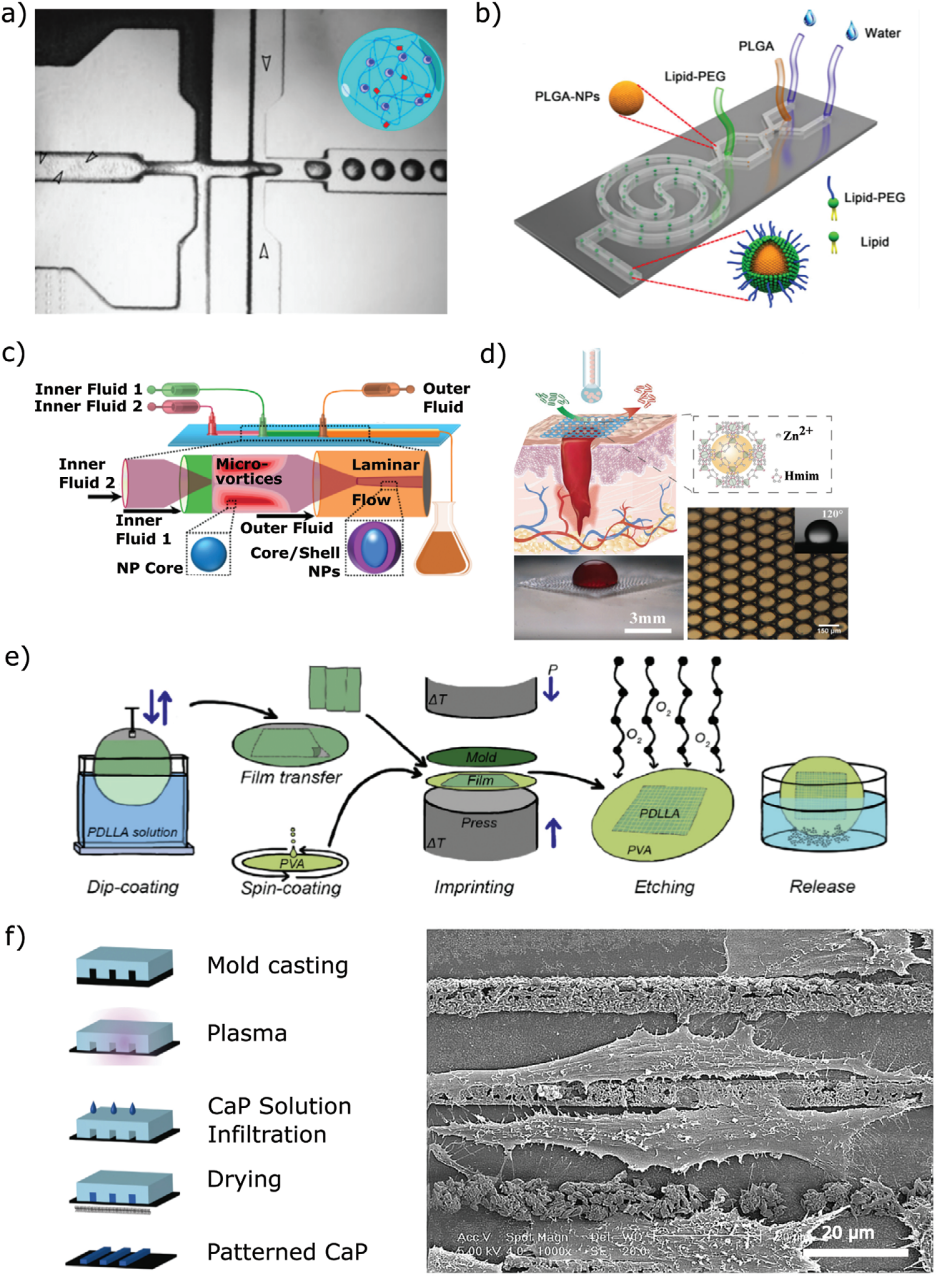
Production of biomaterials on chip. a) Microfluidic encapsulation of stem cells in PVA microgels (Adapted with permission.^[^ ^72^ ^]^ Copyright 2018, Elsevier.); b) high‐throughput synthesis of lipid‐polymer hybrid nanoparticles with tunable diameter (Adapted with permission.^[^ ^96^ ^]^ Copyright 2015, AIP Publishing.); c) production of core–shell nanocomposites by sequential microfluidic nanoprecipitation (Adapted with permission.^[^ ^100^ ^]^ Copyright 2017, American Chemical Society.); d) microfluidic production, microstructure and omniphobic behavior of porous PVA hydrogel membrane containing metal–organic frameworks (Adapted with permission.^[^ ^105^ ^]^ Copyright 2020, Wiley‐VCH GmbH.); e) schematic of micro‐object fabrication by thermal imprinting on soluble sacrificial layer (Adapted with permission. ^[^ ^119^ ^]^ Copyright 2019, Elsevier.); f) left: Schematic of micromolding in capillaries to pattern bioactive ceramics. Right: SEM micrograph of MG63 cells cultured on patterned ceramic for 72 h. Scale bar: 20 µm (Adapted with permission.^[^ ^131^ ^]^ Copyright 2016, Royal Society of Chemistry)

Compared to gelation in bulk emulsions, droplet microfluidics allows the production of highly monodisperse droplets or particles with very fine control of chemical composition and of the number of encapsulated species‐ such as cells‐ per particle, as well as some control of particle shape.^[^ ^78^, ^79^, ^80^ ^]^ This makes it an attractive methodology for the production of gels containing encapsulated cells^[^ ^81^, ^82^, ^83^ ^]^ (**Figure** **1**a) and pharmaceutically relevant compounds or species such as antibodies,^[^ ^84^ ^]^ drugs,^[^ ^85^ ^]^ ions^[^ ^86^ ^]^ or lentiviral vectors.^[^ ^87^ ^]^ In addition to these microgels produced by droplet‐based techniques, coaxial laminar flow in a microfluidic device can be used for the production of hydrogel fibers. For instance, it has been used to produce hydrogel fibers with encapsulated cells,^[^ ^88^, ^89^ ^]^ and self‐assembled polysaccharide fibers^[^ ^90^ ^]^ that in turn have been assembled into fibrous hydrogel films incorporating graphene.^[^ ^91^ ^]^

### 2.2 Production of Nanomaterials on Chip

Nanoparticles, including those used for theranostics or as building blocks of functional biomaterials, are commonly synthesized using microfluidic methods as the controlled and predictable mixing in a microfluidic device gives better control of particle size and composition than bulk methods (Figure 1b,c). As the characteristic mixing time in a microfluidic device is much shorter than in a bulk reactor, the resulting nanoparticles exhibit a narrower size distribution and better batch‐to‐batch consistency.^[^ ^92^, ^93^ ^]^ While the lower reagent volumes inherent to microfluidics mean that smaller amounts of nanoparticles can be produced, for biological applications the increased quality of the particles in terms of controlled size, shape and composition can outweigh this disadvantage. A wide range of substances have been used to produce nanoparticles for these applications, including a range of polymers,^[^ ^94^, ^95^, ^96^ ^]^ multimetallic alloys,^[^ ^97^ ^]^ ceramics, such as hydroxyapatite,^[^ ^98^ ^]^ and silica.^[^ ^99^ ^]^ The potential for extremely rapid mixing of reagents within a microfluidic device has also been exploited for the controlled production of nanoparticles with more complex structure, including core–shell nanocomposites^[^ ^100^ ^]^ and hollow particles.^[^ ^101^ ^]^ Nanoparticles produced or modified using microfluidics have a range of applications, including drug delivery,^[^ ^67^ ^]^ gene delivery,^[^ ^95^ ^]^ and imaging‐assisted cancer therapy.^[^ ^102^ ^]^ For these clinical applications, the batch‐to‐batch consistency and monodispersity of these nanoparticles is an advantage compared to those produced using bulk methods.

### 2.3 Production of Hybrid Biomaterials on Chip

Organic–inorganic hybrid biomaterials such as metal–organic frameworks (MOFs) and composites are an important class of materials for biomedical applications such as drug delivery and bone regeneration. MOFs contain cavities with tunable size which can be used to contain and deliver bioactive molecules, while composites allow the properties of different types of material‐ such as the mechanical properties of a polymer and the bioactivity of a calcium phosphate ceramic‐ to be combined.

Due to the large number of potential combinations of components, the potential of microfluidics for high‐throughput synthesis would be particularly useful for the production of large libraries of candidate materials of this type. Several such materials have been produced in the form of micron‐sized spheres or capsules using droplet microfluidics, which allows the production of spherical monodisperse particles which are well suited to in vivo applications such as drug delivery.^[^ ^103^, ^104^ ^]^ In addition, microfluidic methods have been used to produce MOF‐laden membranes with micropores templated using monodisperse microfluidically generated droplets (Figure 1d),^[^ ^105^ ^]^ and MOF‐containing hydrogel microfibers which exploit the ability of microfluidic spinning to produce fibers with tunable size and composition.^[^ ^106^ ^]^ The category of organic–inorganic hybrid biomaterials produced on chip also includes corresponding composite core–shell microparticles,^[^ ^107^ ^]^ metal‐organic hybrid nanoparticles for biomedical applications such as wound healing and cancer therapy,^[^ ^108^ ^]^ polymer films containing calcium phosphate nanoparticles produced on chip for bone regeneration,^[^ ^109^ ^]^ and bioinks for microfluidic 3D bioprinting containing calcium‐phosphate microparticles for cartilage regeneration.^[^ ^110^ ^]^ All of these materials take advantage of the superior control of particle size and morphology and material composition in microfluidic synthesis compared to bulk methods.

### 2.4 Production of Microstructured and Microscale Biomaterials with Defined Shape on Chip

As well as biomaterials synthesis, on‐chip methods lend themselves to the fabrication of biomaterials with defined microstructure or micron‐scale biomaterial particles with defined geometry. The additive manufacturing of biomaterials using microfluidic print heads^[^ ^111^, ^112^, ^113^ ^]^ is an important example of the use of microfluidics in biomaterials fabrication with a resolution that is often higher than that of other additive manufacturing techniques. Again, the ability of microfluidics to produce gel constructs containing encapsulated cells has been utilized in the expanding field of bioinks for bioprinting.^[^ ^114^, ^115^ ^]^ The improved control of flow in microfluidic print heads allows them to produce 3D scaffolds with defined and variable porosity^[^ ^116^ ^]^ or incorporating gradients of mechanical or (bio)chemical cues,^[^ ^110^ ^]^ and to switch rapidly between different materials to allow the printing of multimaterial constructs.^[^ ^117^ ^]^ In all of these methods, while the final assembly of the construct takes place off‐chip, fluid flow in the microchannels on chip is crucial to obtain the desired control of printing.

Other, non‐microfluidics‐based microfabrication techniques have also been used to produce biomaterials with defined shapes or microstructures for use on chip. Examples include micro‐objects for bottom‐up tissue engineering produced by photolithography^[^ ^118^ ^]^ or thermal imprinting (Figure 1e),^[^ ^119^ ^]^ or microparticles of a range of biomaterials with defined shape, produced by molding or stamping using the particle replication in nonwetting templates (PRINT)^[^ ^120^ ^]^ or step and flash imprint lithography (S‐FIL)^[^ ^121^ ^]^ techniques, or a combination of these two methods.^[^ ^122^ ^]^ These techniques enable the production of microparticles with defined shape which can guide the self‐organization of cells to form a larger‐scale tissue. In addition, microparticles produced using imprinting‐based methods do not contain potentially toxic photoinitiators, while the PRINT technology can be scaled up using roll‐to‐roll continuous manufacturing.

In addition, flow photolithography combines microfluidics with lithography to produce large numbers of anisotropic micro‐objects, including “Janus particles” consisting of two different materials and particles that exhibit temperature‐responsive behavior.^[^ ^123^, ^124^, ^125^, ^126^ ^]^ A recent review by Xue et al. describes the importance of shape in the interaction of these non‐spherical particles with biological systems.^[^ ^127^ ^]^ Finally, direct photolithography methods such as two‐photon polymerization have been used to produce 3D biomaterial structures inside a microfluidic chip as a means to study the combined effects of surface‐bound (topography) and soluble (chemical gradient) cues on cell behavior.^[^ ^128^, ^129^ ^]^

Within the category of on‐chip microfabrication, micropatterning of biomaterials (surfaces) is an often used method for spatial control of cell‐biomaterial interactions. For instance, the method of micromolding in capillaries (MIMIC) involves depositing the material in the channels of a microfluidic chip which is then disassembled to give a pattern of material on the surface of the chip substrate (Figure 1f). MIMIC has been used to pattern both cell‐repellent polymers^[^ ^130^ ^]^ and bioactive or bioinert ceramics,^[^ ^131^ ^]^ enabling simultaneous study of the effect of material chemistry and pattern shape. Other micromolding methods have been used to pattern materials, such as solvent‐based micromolding of biohybrid hydrogels to form microcavities for stem cell culture,^[^ ^132^ ^]^ a combination of electrospinning and micromolding of polycaprolactone scaffolds to study Schwann cell attachment for potential applications in nerve tissue engineering,^[^ ^133^ ^]^ and the use of nanoimprinting to pattern electrospun fiber meshes in order to investigate the effect of topographical libraries on stem cell differentiation.^[^ ^134^ ^]^

### 2.5 New Directions for Production of Biomaterials on Chip

Despite the wide range of biomaterials that have been produced either on chip or using chip‐type microfabricated tools such as molds or print heads, there are still many classes of important biomaterials for which these methods of synthesis have only been explored to a relatively limited extent. For instance, metallic and ceramic bulk or mesoscale (as opposed to nanoparticulate) biomaterials have to our knowledge not yet been synthesized on chip, though, as detailed above, on‐chip methods have been used for micropatterning of ceramics. While ceramic microparticles have been produced using droplet microfluidics, such particles produced on chip have as yet not been used in in vitro biological studies.^[^ ^135^ ^]^

Similarly, metallic alloys that are liquid at low (ambient or close to ambient) temperatures, including eutectic mixtures, are an interesting new class of biomaterials with a wide range of applications.^[^ ^136^, ^137^ ^]^ These metals have been used in a variety of microfluidic systems,^[^ ^138^ ^]^ including in wearable devices for real‐time pulse and movement monitoring^[^ ^139^ ^]^ and as electrodes for in vitro neural stimulation.^[^ ^140^ ^]^ Microfluidic methods have been used to fabricate structures, such as reconfigurable antennae, from such metals for use off‐chip.^[^ ^141^ ^]^ However, to our knowledge, structures made from these low‐melting‐point alloys have yet to be fabricated on chip for use in biomedical applications.

For many different classes of biomaterials, on‐chip methods open up the possibility of combinatorial synthesis to allow researchers to explore a large chemical space.^[^ ^142^ ^]^ Polymer nanoparticles for cancer therapy are one example of an application of such combinatorial biomaterials synthesis on chip.^[^ ^143^ ^]^ In future, similar on‐chip combinatorial methods could be used to explore other regions of chemical space, producing libraries of biomaterials such as ceramics, alloys or organic–inorganic hybrid materials. This could be combined with advances in the fabrication of biomaterials using microfluidic or microfabricated tools such as 3D print heads, in order to bioprint constructs containing combinatorial libraries of materials or with micropatterning techniques to simultaneously explore chemical and topographical/structural parameters in high throughput.

Finally, while on‐chip production has allowed synthesis of a wide range of biomaterials to be achieved, it remains predominantly a research endeavor and has yet to be widely translated into industry or the clinic. Challenges which need to be overcome for this translation to take place successfully include scaling production up to relevant quantities, as well as ensuring that on‐chip methods can be standardized sufficiently to comply with current Good Manufacturing Practices (cGMP).^[^ ^144^, ^145^ ^]^

## 3 Characterization and Screening of Biomaterials

Another application of on‐chip methods to the study of biomaterials is the characterization of their properties and screening of their interactions with a biological system. Large arrays or libraries of biomaterials with different formulations or physical or chemical surface functionalities can be produced, and their interactions with cells can be studied,^[^ ^146^ ^]^ on chip. In a similar manner to that already discussed with respect to biomaterials production, on‐chip screening is a potential solution to the problem of limited time and resources to characterize and screen very large numbers of candidate biomaterials. In addition, all of the candidate materials can be analyzed under identical conditions, increasing the validity of comparisons between them.

For instance, microfluidic chips allow the effect of a range of chemical and mechanical stimuli on cell‐biomaterial interactions to be investigated more easily and with higher throughput than is possible using macroscopic experiments, so a range of devices have been developed to investigate cell‐biomaterial interactions for bone regeneration applications.^[^ ^147^ ^]^ In some cases, the material libraries themselves are produced using on‐chip, particularly microfluidic, methods and remain within the same chip for subsequent screening. In contrast, other studies introduce libraries of materials produced elsewhere, either on a separate chip or using conventional methods, into a chip for screening. Biological systems of increasing complexity, ranging from single cells to whole organisms, have been used to screen biomaterials in microfluidic devices.^[^ ^142^ ^]^ Chips have been developed to investigate specific quantitative aspects of the interaction between cells and materials, such as cell adhesion and migration,^[^ ^148^, ^149^, ^150^, ^151^ ^]^ the ability of materials to stimulate cell proliferation and differentiation,^[^ ^151^, ^152^ ^]^ and antimicrobial activity.^[^ ^153^, ^154^ ^]^ In addition to the biological response to biomaterials, the physical and chemical properties of biomaterials can be studied on chip in the absence of cells, particularly where these properties are different at the biologically relevant micron‐scale compared to in bulk, as in the case of degradability.^[^ ^155^ ^]^

### 3.1 On‐Chip Screening of Biomaterial Library Interactions with Cells

**Figure 2:**
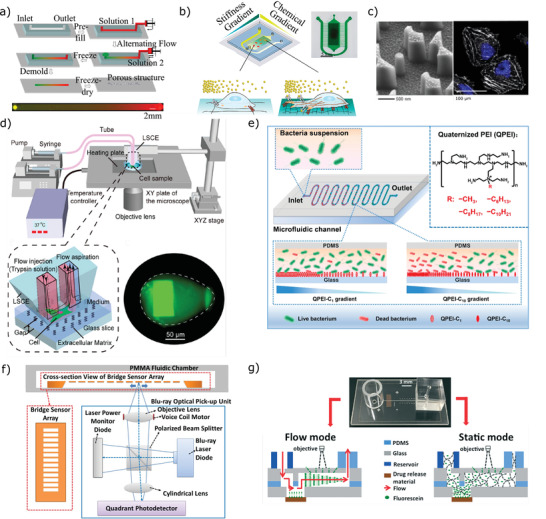
Characterization and Screening of Biomaterials on Chip. a) Microfluidic synthesis of gradient material to investigate cell‐biomaterial interactions (Adapted with permission.^[^ ^157^ ^]^ Copyright 2011, Wiley‐VCH GmbH.); b) crossed on‐chip stiffness and chemical gradient to investigate glioma cell migration (Adapted with permission.^[^ ^158^ ^]^ Copyright 2020, American Chemical Society.); c) response of cells to fabricated nanotopographies on the NanoTopoChip (Adapted with permission.^[^ ^167^ ^]^ Copyright 2017, Elsevier.); d) measurement of cell‐matrix adhesion at single‐cell resolution (Adapted with permission.^[^ ^174^ ^]^ Copyright 2018, American Chemical Society.); e) assessment of antibacterial biomaterial efficacy on a gradient‐functionalized chip (Adapted with permission.^[^ ^154^ ^]^ Copyright 2020, American Chemical Society); f) schematic of a microfluidic device based on a commercial Blu‐ray player to investigate biomaterial degradation Adapted with permission.^[^ ^175^ ^]^ Copyright 2017, Elsevier.); g) microfluidic platform to investigate therapeutic delivery from biomedical device coatings (Adapted with permission.^[^ ^177^ ^]^ Copyright 2017, Royal Society of Chemistry.)

The layer‐by‐layer film libraries produced by Castleberry et al.^[^ ^156^ ^]^ are one example of the first class of on‐chip biomaterials screening mentioned above, where biomaterials are produced and screened on the same chip. In this study bilayer films consisting of polyelectrolytes with differing pH were constructed on chip, and cells cultured directly on these films. Similarly, He et al.^[^ ^157^ ^]^ produced gelatin‐chitosan cross‐gradient composite materials using a microfluidic channel (**Figure** **2**a). After removal of the channel, the resulting porous films were characterized and their effect on the morphology of smooth muscle cells was investigated.

As an alternative to on‐chip biomaterial production and screening, as mentioned above, the materials can be produced elsewhere before incorporation into the chip. One example of this type of study is the polyacrylamide hydrogel with a longitudinal stiffness gradient produced by Dou et al. and incorporated into a chip where a superimposed orthogonal chemical gradient was generated in order to investigate the migration of glioma cells (Figure 2b).^[^ ^158^ ^]^ In contrast to this bulk material, microparticles of gelatin, polyacrylamide and calcium phosphate produced off‐chip using bulk emulsification have been incorporated into multicellular spheroids formed on a chip consisting of an array of non‐adherent microwells.^[^ ^159^, ^160^, ^161^ ^]^ The potential to combine this type of study with existing microfluidic chip designs that include such microwells^[^ ^162^ ^]^ is of great interest for high‐throughput screening of biomaterial particles.

A related class of biomaterial screening studies are those in which microfabrication is used to produce a chip incorporating an arrayed library of surface structural or chemical functionalities in order to study their effect on biological systems. One important example of such a surface displaying a large variety of microtopographies, which were designed using a computational random pattern generator and created using thermal imprinting, is the TopoChip,^[^ ^163^ ^]^ which has been fabricated in a range of materials, including poly(lactic acid) and polystyrene as well as calcium phosphate and TiO_2_ coatings.^[^ ^164^ ^]^ A similar array, the multi‐architecture chip, was fabricated in poly(dimethylsiloxane) (PDMS) by replicating a thermally imprinted polycarbonate master mold and used for studies of neurodifferentiation.^[^ ^165^ ^]^

After initial high‐throughput investigation using the TopoChip, on which the effect of very large arrays of surface properties on cell morphology can be assessed in a densely packed microlibrary by fluorescence microscopy, selected topographies have been reproduced over larger surface areas for use in microwell plates.^[^ ^166^ ^]^ This allows specific topographies of interest to be investigated using other techniques, such as quantitative polymerase chain reaction (qPCR) assays, which typically require more cells. Similar techniques have been used to produce a “nano‐TopoChip” with topographical features on the nanoscale (Figure 2c),^[^ ^167^ ^]^ as well as a “ChemoTopoChip,” which enables simultaneous screening of microtopography and surface chemistry.^[^ ^168^ ^]^

### 3.2 On‐Chip Quantification of Specific Biological Responses to Biomaterials

Certain specific aspects of the interaction of biomaterials with cells and biomolecules have been studied on chips, including biofouling, cell adhesion, differentiation (capacity), and antimicrobial activity. Weiss et al. have used microfluidics to investigate protein fouling on nano‐ and microparticles in flow.^[^ ^169^, ^170^ ^]^ This gave finer control of local flow and shear conditions, which have been shown to affect protein fouling,^[^ ^171^ ^]^ as well as permitting investigations of protein corona formation kinetics over shorter timescales than is possible with static or bulk‐flow experiments. The microfluidic set‐up, with its accurately defined channel dimensions and flow rates, facilitated the design of reproducible standardized experiments, which is important for potential translation of biomaterials research from the lab to the clinic.^[^ ^172^ ^]^

In addition to this study of the interaction of biomaterial particles with biological molecules, microfluidic methods have been developed to investigate the strength of the interaction between cells and biomaterials, which is an important parameter for regenerative medicine applications such as cardiovascular or bone regeneration. For instance, cell adhesion on magnesium alloys or silicone surfaces can be measured in a microenvironment which more accurately mimics the laminar flow and shear conditions in vivo in a small blood vessel.^[^ ^148^, ^173^ ^]^ Other advantages of these microfluidic methods for evaluation of cell‐material adhesion include the ability to perform measurements at single‐cell resolution, as in a recent study using different (ECM‐like) polymeric coatings (Figure 2d),^[^ ^174^ ^]^ as well as the advantages of reproducibility and higher throughput that are a general consequence of microfluidic methods. The De‐Adhesion Number Investigator developed by Hartmann et al.^[^ ^149^, ^150^ ^]^ further reduced the lab space and reagent volume requirements for a microfluidic set‐up by generating the necessary shear flow for cell adhesion measurements in a closed chamber on chip using surface acoustic waves.

The effect of materials on cell migration and proliferation is also important in the field of regenerative medicine. The large number of material properties which can affect this cell behavior makes a high‐throughput platform such as the Rapid Assessment of Migration and Proliferation (RAMP) platform, developed by Dumont et al. and used for studies of Schwann cell proliferation in collagen‐based hydrogels, an attractive option for investigations of cell migration in candidate biomaterials.^[^ ^151^ ^]^ The combination of control of flow shear stress and the ability to make high‐throughput measurements has been used for on‐chip studies of the effect of nanobiocomposite substrate stiffness on the osteogenic differentiation of mesenchymal stromal cells.^[^ ^152^ ^]^ Finally, antimicrobial biomaterials have important clinical applications, and high‐throughput microfluidic platforms have been developed to test the efficacy of these materials, both in discrete chambers^[^ ^153^ ^]^ and using continuous gradients of surface functionalization (Figure 2e).^[^ ^154^ ^]^

### 3.3 On‐Chip Physical and Chemical Characterization of Biomaterials

In addition to the study of interactions between biomaterials and cells/tissues, microfluidic methods have been developed to study a range of intrinsic material properties of interest to biomaterials scientists. Physico‐chemical properties, such as mechanical properties, surface wettability, degradation behavior, and release of incorporated compounds, are all of interest to researchers developing biomaterials.

Microfluidic studies of degradation are meaningful as the environment on a microfluidic chip under flow may replicate the in vivo microenvironment of laminar interstitial or capillary flow more faithfully than bulk fluid in terms of factors such as flow shear stress, which has been shown to affect the degradation rate of, for example, poly(lactic‐*co*‐glycolic acid) (PLGA) scaffolds.^[^ ^155^ ^]^ In addition, a method has been developed to follow the enzymatic degradation of PLGA at different enzyme concentrations on chip using a sensor based on a commercial Blu‐ray player (Figure 2f).^[^ ^175^ ^]^

Biomaterials with the ability to release therapeutic compounds have important clinical applications. As, again, microfluidic devices can provide a more physiologically accurate microenvironment than bulk fluid, such as a macroscopic bioreactor, several microfluidic platforms have been developed in order to investigate the release of drugs from candidate biomaterials. For instance, the release of the anti‐inflammatory drug indomethacin from nanoporous alumina implants was measured in a microfluidic flow system which avoided saturation of the media with dissolved drug molecules, and allowed the short‐term kinetics of drug release to be measured and compared with mathematical models.^[^ ^176^ ^]^ Another, configurable, platform was shown to be capable of measuring the release of fluorescently tagged drug molecules from a variety of nanoporous coatings under either flow or static conditions, thereby mimicking the microenvironment of a drug‐eluting vascular stent and brain implant respectively (Figure 2g). This platform was tested by measuring the release of fluorescein from thin films of nanoporous gold with different morphologies.^[^ ^177^ ^]^

While the mechanical properties of bulk biomaterials are more easily studied using conventional off‐chip methods, a range of microfluidic techniques have been developed to investigate the mechanical properties of microbeads by measuring their deformation as they are forced into confining channels by hydrostatic pressure.^[^ ^178^, ^179^ ^]^ Similarly, while surface wettability is an important property for candidate biomaterials, it is easier to measure using off‐chip methods. However, the extent of hydrophilicity is also important for materials used for the fabrication of microfluidic devices, and can be modified during or after the process of closing the channels of such a device. Therefore, some methods have been developed to measure water contact angle‐ and hence hydrophilicity‐ on chip.^[^ ^180^ ^]^

### 3.4 New Directions for On‐Chip Characterization and Biological Screening of Biomaterials

While some interesting examples of studies exist in which either physico‐chemical properties of biomaterials or their effects on biological system are studied on chip, there are many other properties important to biomaterials scientists for which a chip‐based measurement method would be beneficial but does not yet exist. Off‐chip analysis methods such as mass spectrometry have been coupled to microfluidic chips,^[^ ^181^, ^182^, ^183^ ^]^ but this approach has not yet been applied to the study of on‐chip cell‐material interactions in order to, for example, follow both the degradation of the material and the response of the biological system to it. Similarly, while a range of chemical analysis methods including surface plasmon resonance have been incorporated into microfluidic chips,^[^ ^184^, ^185^ ^]^ these methods have not yet been applied to the study of cell‐biomaterial interactions. Integration of sensors into on‐chip platforms offers the possibility of monitoring evolution of biomaterial properties and biological response in time, and even in a spatially resolved manner, providing information that cannot be obtained using conventional methods. Nevertheless, it remains technically challenging to develop miniaturized platforms that comprise a functional (e.g., clinically relevant) biomaterial, a physiological‐like microenvironment and sensing and read‐out tools, clearly showing a need for further interdisciplinary efforts in this direction.

Regulatory organizations such the United States Food and Drug Administration are moving toward the acceptance of organ‐on‐chip studies (discussed later in this review) as an in vitro alternative to animal models. The assessment of the suitability of candidate biomaterials for use in medical devices is an important potential application for these methods in order to determine the materials’ biocompatibility and bioactivity, while reducing the use of laboratory animals. In order for these methods to be adopted, existing in vitro regulatory tests need to be translated into on‐chip protocols that are acceptable to regulatory agencies. This acceptance depends on the development of on‐chip methods that can sustain cell culture for long enough for the effects of long‐term implantation to become apparent. Even once the relevance of on‐chip results to in vivo performance has been demonstrated, on‐chip studies will necessarily remain an imperfect model of the in vivo environment. Therefore, the implications of any remaining differences between the in vivo and on‐chip environments for the interpretation of on‐chip results also need to be fully understood.^[^ ^186^, ^187^ ^]^ Work also continues on the definition of measurable properties to characterize the efficacy of a material for tissue engineering and regenerative medicine.^[^ ^188^ ^]^ In future, this could help inform the design of on‐chip methods to measure these properties, and allow their designers to make decisions about when it can be safely assumed that a difference between the on‐chip and in vivo environment does not affect the validity of the measurement.

In general, the increased similarity of the (micro)environment on chip to that in vivo could both allow on‐chip experiments to partially replace animal models, and allow studies that are impractical in vivo, such as continuous monitoring using a device such as a mass spectrometer which is too large to be implanted, to be performed in a way that more accurately replicates physiological conditions.

## 4 Biomaterials as Tools for On‐Chip Biology

The final, and perhaps most widespread, use of biomaterials on chip is as tools to develop “organ/tissue/disease on chip” devices for studies of a range of phenomena. In these systems, a biological tissue is modeled in vitro using microfabricated structures, in order to perform experiments that would otherwise require studies in animal models or possibly explanted tissue samples, with their inherent ethical and cost considerations. Additionally, on‐chip models allow the use of human rather than animal cells, which in some respects gives a closer approximation of a human tissue or organ than an animal model.

Biomaterials can fill several different roles in recapitulating the in vivo structural and/or chemical microenvironment. For example, hydrogels such as the widely used Matrigel^[^ ^189^ ^]^ can act as ECM mimics to support tissue or organoid formation.^[^ ^190^, ^191^, ^192^ ^]^ Similarly, a calcium phosphate‐based biomaterial can mimic the inorganic part of the bone ECM.^[^ ^193^, ^194^, ^195^ ^]^ 2‐dimensional (2D) porous membranes, made of biocompatible materials that allow adherent cell growth, can be used to mimic physiological barriers such as the blood‐brain barrier^[^ ^196^, ^197^ ^]^ or endothelial barrier^[^ ^198^, ^199^ ^]^ and establish a compartmentalized tissue/organ architecture. Moreover, such membranes can be used to support cell growth and potentially transfer mechanical stimuli such as stretching to the cells.^[^ ^200^, ^201^, ^202^, ^203^ ^]^ Alternatively, biocompatible low‐attachment materials with either 3D or quasi‐2D structure can be used as a platform to confine and protect cell aggregates, inducing them to assemble into 3D structures such as spheroids or organoids while keeping them in defined positions for repeated observation.^[^ ^204^, ^205^, ^206^ ^]^ A hydrogel biomaterial can also be used to fabricate the entire device, with microfabricated channels acting as model vasculature.^[^ ^207^, ^208^ ^]^ Finally, soluble or degradable biomaterials can act as a sacrificial scaffold which is then removed to produce a free‐standing artificial tissue or organ consisting entirely of cells and the ECM they have produced.^[^ ^209^ ^]^

**Figure 3:**
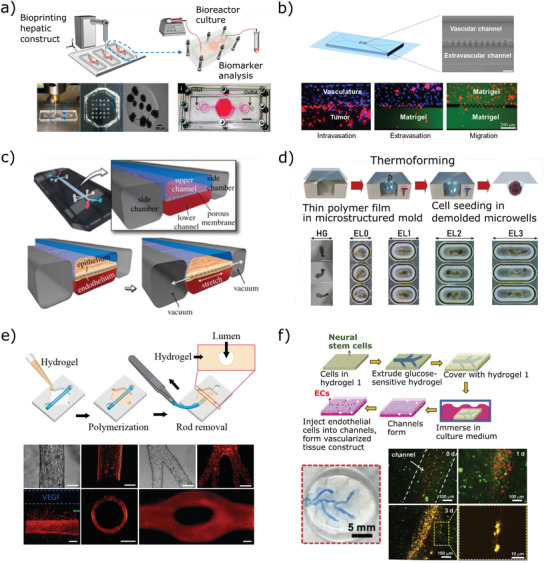
Biomaterials as tools for on‐chip biology. a) A liver‐on‐chip construct based on bioprinted hepatic spheroids (Adapted with permission.^[^ ^224^ ^]^ Copyright 2016, IOP Publishing); b) an on‐chip model of metastasis (Adapted with permission.^[^ ^228^ ^]^ Copyright 2019, American Chemical Society.); c) a human lung‐on‐chip device which uses vacuum actuation to replicate the action of breathing (Adapted with permission.^[^ ^240^ ^]^ Copyright 2013, Nature Publishing Group.); d) fabrication of anisotropic microwells to induce differentiation in embryoid bodies (top) and images of cell aggregates in the differently shaped microwells and in hanging drops (bottom) (Adapted with permission.^[^ ^247^ ^]^ Copyright 2020, Wiley‐VCH GmbH.); e) schematic of the production of hydrogel lumens using PDMS rods and the LumeNEXT method (top) and endothelial cell‐lined lumens produced using this method (bottom) (Adapted with permission.^[^ ^208^ ^]^ Copyright 2016, Wiley​‐VCH GmbH.) Production of a vascularized neural tissue construct using a glucose‐sensitive hydrogel to produce endothelialized channels (Adapted with permission.^[^ ^260^ ^]^ Copyright 2017, Elsevier.)

The similarity in scale of microfluidic channels to body fluid‐containing/‐transporting lumen such as blood or lymphatic vessels/capillaries or kidney tubules has enabled the field of “organ on chip” studies.^[^ ^210^, ^211^ ^]^ A range of tissues and organs have been modeled on chip through the use of biomaterials, including the placenta,^[^ ^212^ ^]^ liver (**Figure** **3**a),^[^ ^213^ ^]^ intestine^[^ ^214^ ^]^ and cartilage.^[^ ^215^ ^]^ Similar individual models have been combined in “body‐on‐chip” devices in which biomaterials are used to replicate the specific microenvironments of several different organs or tissues which are in communication with each other.^[^ ^216^, ^217^, ^218^ ^]^ While the earliest of such devices contained 2D cultures,^[^ ^219^ ^]^ biomaterials are often necessary to enable 3D cell culture that more accurately replicates the in vivo environment.^[^ ^220^ ^]^

### 4.1 Biomaterials as ECM Mimics

In on‐chip platforms where a biomaterial is used to mimic ECM, hydrogels play an important role. The gas, water and solute permeability of these hydrated polymer networks allows them to serve as ECM substitutes with defined properties, including cell adhesivity, stiffness, and degradation dynamics, to support cell attachment, migration and self‐organization, such as the formation, growth, differentiation and maturation of organoids.^[^ ^221^, ^222^, ^223^, ^224^ ^]^ Therefore, they have been used as ECM models to study processes including angiogenesis,^[^ ^225^ ^]^ osteoblast migration,^[^ ^30^ ^]^ and cancer metastasis (Figure 3b).^[^ ^226^, ^227^, ^228^ ^]^ In addition, a wide range of techniques, including stereolithography,^[^ ^229^ ^]^ soft lithography,^[^ ^230^ ^]^ and microfluidics,^[^ ^117^ ^]^ combined with various chemical crosslinking strategies, have been used to produce patterned hydrogel scaffolds for 3D cell culture. The incorporation of chemical, mechanical or structural patterning allows better mimicry of the in vivo microenvironment, thus giving improved control over cell fate compared to an isotropic and homogeneous hydrogel.^[^ ^231^ ^]^

While 3D hydrogels such as Matrigel are useful as models of the ECM in many tissue types, they are not universally applicable. For instance, the basal lamina in barrier tissues is effectively a 2D ECM and can therefore be modeled by a 2D biomaterial membrane rather than a 3D hydrogel matrix^[^ ^232^ ^]^—for instance, collagen has been used to model the basal lamina of the placenta^[^ ^233^ ^]^ or colon.^[^ ^234^ ^]^ Membranes made from other bioactive polymers such as peptide amphiphiles and polycaprolactone/chitosan blends have been used to produce microfluidic models of brain tissue interfaces^[^ ^235^ ^]^ and to monitor transendothelial hydraulic resistance.^[^ ^236^ ^]^

Similarly, in bone‐on‐chip studies, relatively soft hydrogels are a poor mimic of native bone matrix. Therefore, different biomaterials have been used to mimic the hard, mineralized ECM of bone, including fibrin incorporating hydroxyapatite,^[^ ^237^ ^]^ mineralized collagen,^[^ ^227^ ^]^ and tightly packed calcium phosphate microbeads,^[^ ^194^ ^]^ as well as decellularized human^[^ ^238^ ^]^ or animal^[^ ^239^ ^]^ bone. These models have been used to investigate cancer metastasis into bone^[^ ^227^, ^239^ ^]^ or bone angiogenesis,^[^ ^193^ ^]^ to investigate the mechanotransduction of osteocytes,^[^ ^194^ ^]^ and to study the effect of mechanical stimulation on bone formation over timescales of multiple years.^[^ ^238^ ^]^

### 4.2 Biomaterial Membranes for Cell Culture

The second important role of biomaterials in organ/disease‐on‐chip devices is as a membrane to take over the structural functions of 2D ECM for support and mechanotransduction, without mimicking its chemical properties. A review by Pasman et al.^[^ ^200^ ^]^ details the importance of porous polymeric membranes to separate “blood vessel” and “organ” compartments in these devices, describing the effects of the different membrane materials used, their properties, and the methods used to prepare them. A range of different materials can be used depending on the desired properties. PDMS is particularly popular as it is easily processed, flexible, transparent, gas permeable, and its mechanical properties and hydrophobicity are tunable by changing the ratio of PDMS base to curing agent and by surface plasma treatment respectively.^[^ ^240^ ^]^ However, its propensity to absorb small molecules can be problematic for some studies.^[^ ^241^ ^]^ In contrast, polymers such as polycarbonate^[^ ^214^ ^]^ and poly(ethylene terephthalate),^[^ ^242^ ^]^ while biocompatible and inert, are much stiffer than PDMS so are unsuitable for applications where the membrane must be flexible.

In addition to simply acting as a substrate for cell culture at the interface between two compartments, if these membranes are fabricated from a sufficiently elastic biomaterial such as PDMS they can be stretched and/or bent in order to apply mechanical forces to the cells to mimic biological processes such as breathing (Figure 3c)^[^ ^201^, ^203^, ^240^ ^]^ and gut peristalsis^[^ ^202^, ^243^ ^]^ or to measure the mechanical properties of monolayers of cells.^[^ ^244^ ^]^

### 4.3 Biomaterials for Cell Aggregation and Confinement

In contrast to the biomaterials for adherent cell culture described above, low‐attachment microwells are used to induce cells to aggregate into 3D structures such as spheroids, embryoid bodies, or organoids in response to confinement. In this case, the biomaterial functions as a protective niche to allow aggregation and self‐organization. These microwell arrays can be produced either from PDMS^[^ ^245^ ^]^ or from hydrogels such as agarose.^[^ ^205^ ^]^

Arrays of these microwells have also been produced from polymeric membranes using microfabrication techniques, such as microthermoforming.^[^ ^246^ ^]^ These membrane‐based confinement devices have been used to study morphogenesis in early embryonic development (Figure 3d).^[^ ^247^ ^]^ Moreover, thermoformed membrane‐based microwell devices have been used to culture pancreatic islets.^[^ ^248^, ^249^ ^]^ A similar membrane‐based carrier will allow extrahepatic implantation of these islets in the not too distant future. It should be noted that, like the polymer membranes discussed in section 4.2, the polymers used for the fabrication of microwell arrays are normally technical, bioinert or biodegradable^[^ ^250^ ^]^ polymers that do not mimic the chemical characteristics of ECM.

### 4.4 Hydrogels as Device Materials

An interesting subset of microfluidic devices incorporating biomaterials is those where the entire device is fabricated from a hydrogel. One of the first examples of this is the microfluidic channels made from alginate hydrogel by Cabodi and co‐workers in 2005.^[^ ^207^ ^]^ These microfluidic devices made from hydrogels are of particular interest for the study of blood vessels,^[^ ^251^ ^]^ which is important both in the field of regenerative medicine, as part of efforts to produce engineered tissues with functional vasculature,^[^ ^252^ ^]^ and for the production of more realistic in vitro models with lower vessel wall stiffness.^[^ ^253^ ^]^

One method for producing blood vessel‐like microfluidic channels in hydrogels is the LumeNext approach developed by Beebe and co‐workers in 2015,^[^ ^208^ ^]^ in which an ECM‐like hydrogel is polymerized around a PDMS rod which is then removed (Figure 3e). This method has been used to model microvessels,^[^ ^254^ ^]^ patient‐specific blood vessels,^[^ ^255^ ^]^ and mammary ducts.^[^ ^256^ ^]^ Other fabrication methods used to produce these channels in hydrogels include 3D printing,^[^ ^257^ ^]^ gelatin sacrificial molding,^[^ ^258^ ^]^ and laser‐based degradation.^[^ ^259^ ^]^

### 4.5 Biomaterials as Temporary Support

A final category of biomaterials in the production of microfabricated chips for in vitro organ/disease‐on‐chip models is those which support the formation of a biological tissue inside a microdevice, but then are removed to produce a fully biological tissue. This application requires biomaterials which maintain their integrity for long enough to support the formation of a free‐standing tissue with the desired architecture, but then can be degraded or removed either over time (via biodegradation or slow dissolution) or in response to a physical or chemical stimulus that does not affect the integrity of the artificial tissue. A range of biomaterials have been used for this, including glucose‐sensitive hydrogels to fabricate vascular channels (Figure 3f),^[^ ^260^ ^]^ biodegradable polymers in 3D bioprinting,^[^ ^261^ ^]^ and thermoresponsive, electroresponsive or pH‐responsive polymers to allow the detachment of free‐standing anisotropic cell sheets.^[^ ^262^, ^263^, ^264^ ^]^ One recent application of this type of technique is the AngioChip scaffold, which consists of a hydrolytically degradable elastomer network containing a range of micro‐ and nanopores to allow cell migration and biomolecular exchange. These highly permeable scaffolds, which degrade over an extended timescale, support ECM formation and the growth of engineered vascularized cardiac and hepatic tissue both in bioreactors and in vivo.^[^ ^265^ ^]^ For a much shorter degradation timescale, temporary chitosan membranes which can be washed away using a weak acetic acid solution have been used as supports for membrane‐free blood‐brain barrier models.^[^ ^266^ ^]^

### 4.6 New Directions for Biomaterials as Tools for Organ‐on‐Chip and Disease‐on‐Chip Models

While biomaterials are important in the production of organ/tissue/disease‐on‐chip devices, so far most efforts have concentrated on the production of a biomimetic microenvironment for cells that replicates the natural conditions in a living organism. In these devices the chosen biomaterial either mimics part of the natural cell niche (such as ECM), or is chosen so as to support tissue or organoid culture without perturbing it (as in the use of porous polymeric membranes in model barrier tissues). Currently, the environment of an implanted biomaterial can only be modeled using animals. Using conventional techniques to study a wide range of different materials requires the use of a large number of animals, as only a limited number of samples can be implanted per animal. Implantable chips can allow multiple material samples to be tested in one animal, reducing the number of animals required.^[^ ^267^ ^]^

In future, organ‐on‐chip devices could be developed that model the in vivo cell‐biomaterial interaction when a biomaterial is used in medical treatment, as part of an implanted medical device or for regenerative therapy.^[^ ^186^ ^]^ One of the first examples of this, to our knowledge, is the “tooth on a chip” device to evaluate the response of dental pulp cells to biomaterials,^[^ ^268^ ^]^ partially replicating the environment of a dental implant. However, in order to accurately recapitulate the biological environment of an implant, organ‐on‐chip systems would need to be capable of modeling the inflammatory and immune response to the biomaterial. While on‐chip models of the immune response are an active field of research,^[^ ^269^, ^270^ ^]^ models of the immune response to candidate biomaterials are still at a relatively early stage and do not yet include an accurate model of the implant environment including features such as foreign body immune response and innervation.^[^ ^271^, ^272^ ^]^ It should be noted that this challenge of incorporating multiple (including systemic) in vivo‐like responses to a biomaterial is valid for all in vitro models, not only those on chip. However, because of the advantages of miniaturized models discussed so far, they offer opportunities to efficiently overcome this challenge.

## 5 Future Outlook

In addition to the trends in specific areas of biomaterials‐on‐chip research, which have been discussed in previous sections, there are certain trends which are apparent across the whole of the field. In particular, these are increased scale and automation. Taken together, in future they will allow both the rapid generation of large amounts of biomaterials‐related data and the rapid production of significant quantities of customized biomaterials for research and clinical applications.

The production of materials at scale using microfluidics is possible, despite the low volume inherent to microfluidic systems, by massively parallelizing these systems. For example, a single device has been produced incorporating more than 10000 droplet generators, allowing poly(caprolactone) microparticles to be produced at the rate of hundreds of grams per hour.^[^ ^273^ ^]^ Parallel droplet generators have also been used to produce large quantities of hydrogels for drug delivery,^[^ ^274^ ^]^ while other parallel microfluidic devices have been used to produce polymeric nanoparticles.^[^ ^275^ ^]^ While the mass production of hydrogel fibers using parallelized microfluidic spinning has been suggested,^[^ ^144^, ^276^ ^]^ to our knowledge it has not yet been demonstrated. The possibility of parallelization needs to be further exploited to increase the throughput of production of a variety of biomaterials in order to bring on‐chip technology closer to clinical applications while retaining the high degree of control over the production process which it affords.

As well as the advances in materials production on chip detailed above, the scale‐up of on‐chip biomaterials research would be assisted by the sustainable mass production of the chips required. A recent study details the use of a range of microfabrication technologies to produce 3D molds which in turn can be used to produce chips from thermoplastic biomaterials for organ‐on‐chip applications.^[^ ^277^ ^]^ This scaling up of organ chip production could also be combined with the use of sustainable and bioactive materials, such as poly(lactic acid), to replace the technical, inert elastomers commonly used in organ‐on‐chip development.^[^ ^278^ ^]^ By developing such methods, the specialized fabrication equipment required for on‐chip research will become less important, which in turn will aid the further adoption of these techniques in the field of biomaterials.

Another important future direction for on‐chip synthesis and studies of biomaterials is automation, to allow the generation and analysis of very large material libraries, and in turn similarly large sets of data on cell‐biomaterial interactions. This advance can be considered by looking at each step in the “cycle” of biomaterials library production, high‐throughput screening and in‐depth characterization in turn.

On‐chip methods allow potential high‐throughput studies of biomaterials with various bulk or surface modifications or functionalizations concerning chemistry, material micro‐ or nanostructure, or micro‐ or nanotopography. The last of these can either be explicitly defined via microfabrication techniques, or implicit in the form of roughness derived from the synthesis or post‐synthetic processing of the material. To fully unlock this potential, the synthesis of large libraries of materials with different combinations of these properties needs to be automated in a manner analogous to recent developments in automated chemical synthesis.^[^ ^279^, ^280^, ^281^ ^]^ While arrays of procedurally generated topographies such as the TopoChip^[^ ^163^ ^]^ and ChemoTopoChip^[^ ^168^ ^]^ are an important step in this direction, true high‐throughput biomaterial screening requires the bulk composition and micro/nanostructure as well as the surface chemistry and topography of the materials to vary across the library in a similar fashion to existing DNA or protein microarrays. Work on the generation of libraries of ceramic microparticles with variable composition continues in our laboratory, but automation has yet to be applied to the generation of similar libraries of microstructured polymeric, metallic or hybrid biomaterials.

As well as synthesis and functionalization of biomaterials libraries, in future, the measurement of their properties on chip could be automated, including the range of biological and non‐biological properties for which on‐chip measurements are described in this review. As several of these methods rely on observing deformation of either the chip or the materials, image processing algorithms could be used to automate the generation of data from these observations.^[^ ^282^, ^283^ ^]^ Other experiments rely on chemical analysis, which in turn can be automated, such as by coupling the chip to a mass spectrometer^[^ ^284^ ^]^ or other analytical equipment. The transport of cells and liquids within the chip could be controlled automatically by incorporating sensors and actuators in a closed‐loop feedback arrangement.^[^ ^285^, ^286^ ^]^

A range of other workflows important in on‐chip biology can be automated through the use of robotic manipulation and dispensing equipment, for example to dispense cell and microsized biomaterial suspensions for the production of cell‐biomaterial aggregates or corresponding organoids.^[^ ^287^ ^]^ Automation has also been applied to the operation and monitoring of organ‐on‐chip systems,^[^ ^43^ ^]^ including making fluidic couplings between multiple such chips in a body‐on‐chip arrangement,^[^ ^288^ ^]^ though the production of organs on a chip has not yet been automated.^[^ ^289^ ^]^

**Figure 4:**
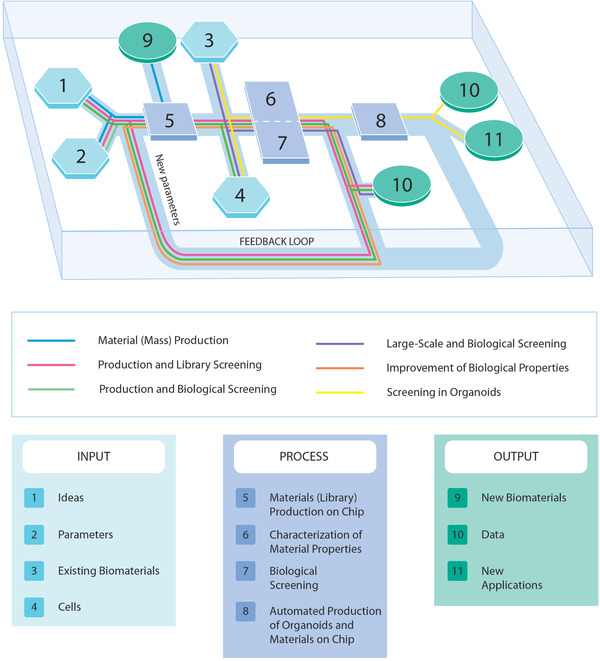
An illustration of a hypothetical automated "research programme on a chip" to produce libraries of biomaterials, characterize the materials and their interactions with cells, and culture cell aggregates and organoids in contact with biomaterials, in order both to produce new materials for biomedical applications and to inform the next iteration of materials library production and screening.

Advances in on‐chip studies of biomaterials have allowed experiments that blend formerly separate fields from materials science to cell biology—these experiments at the interface between fields have enhanced our understanding of cell‐material interactions. In future, it is possible to imagine a single automated workflow (**Figure** **4**) that could generate a library of biomaterials with chemical and physical properties varying within defined parameters, introduce them into a microfabricated screening device or one that replicates in vivo cell‐material interactions, and perform experiments. An artificially intelligent “robot scientist”^[^ ^290^ ^]^ could then analyze the data generated by these experiments and use it to compute parameters for the next generation of biomaterials libraries or the design of further experiments. Iteration over this loop could rapidly produce tailored biomaterials for vital biomedical applications.

## Conflict of Interest

The authors declare no conflict of interest.

## References

[1] K. D. Wise , K. Najafi , Science 1991, 254, 1335. 1962192 10.1126/science.1962192

[2] D. B. Weibel , W. R. DiLuzio , G. M. Whitesides , Nat. Rev. Microbiol. 2007, 5, 209. 17304250 10.1038/nrmicro1616

[3] D. R. Reyes , D. Iossifidis , P. A. Auroux , A. Manz , Anal. Chem. 2002, 74, 2623. 12090653 10.1021/ac0202435

[4] M. L. Kovarik , D. M. Ornoff , A. T. Melvin , N. C. Dobes , Y. Wang , A. J. Dickinson , P. C. Gach , P. K. Shah , N. L. Allbritton , Anal. Chem. 2013, 85, 451. 23140554 10.1021/ac3031543 PMC3546124

[5] S. F. Berlanda , M. Breitfeld , C. L. Dietsche , P. S. Dittrich , Anal. Chem. 2021, 93, 311. 33170661 10.1021/acs.analchem.0c04366

[6] H. Shi , K. Nie , B. Dong , M. Long , H. Xu , Z. Liu , Chem. Eng. J. 2019, 361, 635.

[7] K. S. Burke , D. Parul , M. J. Reddish , R. B. Dyer , Lab Chip 2013, 13, 2912. 23760106 10.1039/c3lc50497b PMC3733270

[8] H. Sun , T. Olsen , J. Zhu , J. Tao , B. Ponnaiya , S. A. Amundson , D. J. Brenner , Q. Lin , RSC Adv. 2015, 5, 4886. 25883782 10.1039/C4RA13356K PMC4394375

[9] J. Kai , A. Puntambekar , N. Santiago , S. H. Lee , D. W. Sehy , V. Moore , J. Han , C. H. Ahn , Lab Chip 2012, 12, 4257. 22914859 10.1039/c2lc40585g

[10] R. Nasiri , A. Shamloo , S. Ahadian , L. Amirifar , J. Akbari , M. J. Goudie , K. Lee , N. Ashammakhi , M. R. Dokmeci , D. Di Carlo , A. Khademhosseini , Small 2020, 16, 2000171. 10.1002/smll.202000171 32529791

[11] Y. Yang , S. Le Gac , L. W. M. M. Terstappen , H. S. Rho , Electrophoresis 2018, 39, 548. 29193175 10.1002/elps.201700351

[12] B. M. Liszka , H. S. Rho , Y. Yang , A. T. M. Lenferink , L. W. M. M. Terstappen , C. Otto , RSC Adv. 2015, 5, 49350.

[13] D. Huh , G. A. Hamilton , D. E. Ingber , Trends Cell Biol. 2011, 21, 745. 22033488 10.1016/j.tcb.2011.09.005 PMC4386065

[14] V. I. Chin , P. Taupin , S. Sanga , J. Scheel , F. H. Gage , S. N. Bhatia , Biotechnol. Bioeng. 2004, 88, 399. 15486946 10.1002/bit.20254

[15] G. M. Whitesides , Nature 2006, 442, 368. 16871203 10.1038/nature05058

[16] T. M. Squires , S. R. Quake , Rev. Mod. Phys. 2005, 77, 977.

[17] E. M. Purcell , Am. J. Phys. 1977, 45, 3.

[18] H. A. Stone , A. D. Stroock , A. Ajdari , Annu. Rev. Fluid Mech. 2004, 36, 381.

[19] A. Günther , K. F. Jensen , Lab Chip 2006, 6, 1487. 17203152 10.1039/b609851g

[20] J. Cui , M. Björnmalm , K. Liang , C. Xu , J. P. Best , X. Zhang , F. Caruso , Adv. Mater. 2014, 26, 7295. 25209733 10.1002/adma.201402753

[21] J. Y. Park , S. J. Yoo , C. M. Hwang , S. H. Lee , Lab Chip 2009, 9, 2194. 19606296 10.1039/b822006a

[22] X. Mei , K. Middleton , D. Shim , Q. Wan , L. Xu , Yu‐H V Ma , D. Devadas , N. Walji , L. Wang , E. W. K. Young , L. You , Integr. Biol. 2019, 11, 119. 10.1093/intbio/zyz008 31125041

[23] C. M. Sakolish , G. J. Mahler , RSC Adv. 2017, 7, 4216.

[24] N. Picollet‐D'hahan , M. E. Dolega , L. Liguori , C. Marquette , S. L.e Gac , X. Gidrol , D. K. Martin , Trends Biotechnol. 2016, 34, 757. 27497676 10.1016/j.tibtech.2016.06.012

[25] R. Gómez‐Sjöberg , A. A. Leyrat , D. M. Pirone , C. S. Chen , S. R. Quake , Anal. Chem. 2007, 79, 8557. 17953452 10.1021/ac071311w

[26] M. C. Liu , D. Ho , Y. C. Tai , Sens. Actuators, B 2008, 129, 826.

[27] H. Somaweera , A. Ibraguimov , D. Pappas , Anal. Chim. Acta 2016, 907, 7. 26802998 10.1016/j.aca.2015.12.008

[28] N. Lin , X. Zhou , X. Geng , C. Drewell , J. Hübner , Z. Li , Y. Zhang , M. Xue , U. Marx , B. Li , Sci. Rep. 2020, 10, 8879. 32483208 10.1038/s41598-020-65817-0 PMC7264205

[29] L. Mathur , M. Ballinger , R. Utharala , C. A. Merten , Small 2020, 16, 1904321. 10.1002/smll.201904321 31747127

[30] N. Movilla , C. Borau , C. Valero , J. M. García‐Aznar , Bone 2018, 107, 10. 29107125 10.1016/j.bone.2017.10.025

[31] Y. Shin , S. Lim , J. Kim , J. S. Jeon , H. Yoo , B. Gweon , Lab Chip 2019, 19, 3664. 31565711 10.1039/c9lc00352e

[32] M. Shang , R. H. Soon , C. T. Lim , B. L. Khoo , J. Han , Lab Chip 2019, 19, 369. 30644496 10.1039/c8lc00970h

[33] S.‐Y. Teh , R. Lin , L.‐H. Hung , A. P. Lee , Lab Chip 2008, 8, 198. 18231657 10.1039/b715524g

[34] T. S. Kaminski , O. Scheler , P. Garstecki , Lab Chip 2016, 16, 2168. 27212581 10.1039/c6lc00367b

[35] G. S. Du , J. Z. Pan , S. P. Zhao , Y. Zhu , J. M. J. Den Toonder , Q. Fang , Anal. Chem. 2013, 85, 6740. 23786644 10.1021/ac400688f

[36] M. Courtney , X. Chen , S. Chan , T. Mohamed , P. P. N. Rao , C. L. Ren , Anal. Chem. 2017, 89, 910. 27959505 10.1021/acs.analchem.6b04039

[37] Š. Selimović , H. Kaji , H. Bae , A. Khademhosseini , in Microfluidic Cell Culture Systems, Elsevier, New York 2019, pp. 31.

[38] J. Wu , G. Zheng , L. M. Lee , Lab Chip 2012, 12, 3566. 22878811 10.1039/c2lc40517b

[39] S. Chen , M. H. Shamsi , J. Micromech. Microeng. 2017, 27, 083001.

[40] Z. Liao , Y. Zhang , Y. Li , Y. Miao , S. Gao , F. Lin , Y. Deng , L. Geng , Biosens. Bioelectron. 2019, 126, 697. 30544083 10.1016/j.bios.2018.11.032

[41] Z. Zhu , O. Frey , N. Haandbaek , F. Franke , F. Rudolf , A. Hierlemann , Sci. Rep. 2015, 5, 17180. 26608589 10.1038/srep17180 PMC4660434

[42] S. M. Bonk , M. Stubbe , S. M. Buehler , C. Tautorat , W. Baumann , E. D. Klinkenberg , J. Gimsa , Biosensors 2015, 5, 513. 26263849 10.3390/bios5030513 PMC4600170

[43] Y. S. Zhang , J. Aleman , Su R Shin , T. Kilic , D. Kim , S. A. Mousavi Shaegh , S. Massa , R. Riahi , S. Chae , N. Hu , H. Avci , W. Zhang , A. Silvestri , A. Sanati Nezhad , A. Manbohi , F. De Ferrari , A. Polini , G. Calzone , N. Shaikh , P. Alerasool , E. Budina , J. Kang , N. Bhise , J. Ribas , A. Pourmand , A. Skardal , T. Shupe , C. E. Bishop , M. R. Dokmeci , A. Atala , A. Khademhosseini , Proc. Natl. Acad. Sci. 2017, 114, E2293. 28265064 10.1073/pnas.1612906114 PMC5373350

[44] P. Joshi , M. Y. Lee , Biosensors 2015, 5, 768. 26694477 10.3390/bios5040768 PMC4697144

[45] D. Sheyn , D. Cohn‐Yakubovich , S. Ben‐David , S. De Mel , V. Chan , C. Hinojosa , N. Wen , G. A. Hamilton , D. Gazit , Z. Gazit , Microfluid. Nanofluid. 2019, 23, 99. 32296299 10.1007/s10404-019-2261-7 PMC7158882

[46] K. Middleton , S. Al‐Dujaili , X. Mei , A. Günther , L. You , J. Biomech. 2017, 59, 35. 28552413 10.1016/j.jbiomech.2017.05.012

[47] W. Y. Sim , S. W. Park , S. H. Park , B. H. Min , S. R. Park , S. S. Yang , Lab Chip 2007, 7, 1775. 18030400 10.1039/b712361m

[48] D.i. Chouhan , S. Mehrotra , O. Majumder , B. B. Mandal , ACS Biomater. Sci. Eng. 2019, 5, 92. 33405874 10.1021/acsbiomaterials.8b00240

[49] J. J. Rice , M. M. Martino , L. De Laporte , F. Tortelli , P. S. Briquez , J. A. Hubbell , Adv. Healthcare Mater. 2013, 2, 57. 10.1002/adhm.201200197 23184739

[50] O. S. Fenton , K. N. Olafson , P. S. Pillai , M. J. Mitchell , R. Langer , Adv. Mater. 2018, 30, 1705328. 10.1002/adma.201705328 PMC6261797 29736981

[51] R. M. Raftery , D. P. Walsh , L. Blokpoel Ferreras , I. Mencía Castaño , G. Chen , M. LeMoine , G. Osman , K. M. Shakesheff , J. E. Dixon , F. J. O'Brien , Biomaterials 2019, 216, 119277. 31252371 10.1016/j.biomaterials.2019.119277

[52] L. Moroni , J. R. De Wijn , C. A. Van Blitterswijk , Biomaterials 2006, 27, 974. 16055183 10.1016/j.biomaterials.2005.07.023

[53] S. J. Hollister , R. D. Maddox , J. M. Taboas , Biomaterials 2002, 23, 4095. 12182311 10.1016/s0142-9612(02)00148-5

[54] N. D. Evans , C. Minelli , E. Gentleman , V. LaPointe , S. N. Patankar , M. Kallivretaki , X. Chen , C. J. Roberts , M. M. Stevens , Eur. Cells Mater. 2009, 18, 1. 10.22203/ecm.v018a01 19768669

[55] J. S. Park , J. S. Chu , A. D. Tsou , R. Diop , Z. Tang , A. Wang , S. Li , Biomaterials 2011, 32, 3921. 21397942 10.1016/j.biomaterials.2011.02.019 PMC3073995

[56] N. Davison , X. Luo , T. Schoenmaker , V. Everts , H. Yuan , F. Barrère‐de Groot de Bruijn , Eur. Cells Mater. 2014, 27, 281. 10.22203/ecm.v027a20 24733686

[57] N. Davison , J. Su , H. Yuan , J. van den Beucken , J. de Bruijn , F. Barrère‐de Groot , Eur. Cells Mater. 2015, 29, 314. 10.22203/ecm.v029a24 26091730

[58] J. Zhang , X. Luo , D. Barbieri , A. M. C. Barradas , J. D. De Bruijn , C. A. Van Blitterswijk , H. Yuan , Acta Biomater. 2014, 10, 3254. 24681376 10.1016/j.actbio.2014.03.021

[59] C. Danoux , L. Sun , G. Koçer , Z. T. Birgani , D. Barata , J. Barralet , C. Van Blitterswijk , R. Truckenmüller , P. Habibovic , Adv. Mater. 2016, 28, 1803. 26689847 10.1002/adma.201504589

[60] L. Sun , C. B. Danoux , Q. Wang , D. Pereira , D. Barata , J. Zhang , V. Lapointe , R. Truckenmüller , C. Bao , X. Xu , P. Habibovic , Acta Biomater. 2016, 42, 364. 27318269 10.1016/j.actbio.2016.06.018

[61] V. P. Galván‐Chacón , P. Habibovic , Adv. Healthcare Mater. 2017, 6, 1601478. 10.1002/adhm.201601478 28544743

[62] M. Macgregor , R. Williams , J. Downes , A. Bachhuka , K. Vasilev , Materials 2017, 10, 1081. 28906470 10.3390/ma10091081 PMC5615735

[63] Á. J. Leite , M. B. Oliveira , S. G. Caridade , J. F. Mano , Adv. Funct. Mater. 2017, 27, 1701219.

[64] D. Lopes , C. Fernandes , J. M. Nóbrega , S. G. Patrício , M. B. Oliveira , J. F. Mano , Acta Biomater. 2019, 96, 222. 31255663 10.1016/j.actbio.2019.06.047

[65] E. Guermani , H. Shaki , S. Mohanty , M. Mehrali , A. Arpanaei , A. K. Gaharwar , A. Dolatshahi‐Pirouz , Sci. Rep. 2016, 6, 30445. 27465860 10.1038/srep30445 PMC4964594

[66] K. Parratt , J. Jeong , P. Qiu , K. Roy , Lab Chip 2017, 17, 2861. 28726912 10.1039/c7lc00451f PMC5577978

[67] L. Zhang , Q. Chen , Y. Ma , J. Sun , ACS Appl. Bio Mater. 2020, 3, 107. 10.1021/acsabm.9b00853 35019430

[68] S. Xin , J. Dai , C. A. Gregory , A. Han , D. L. Alge , Adv. Funct. Mater. 2020, 30, 1907102. 38213754 10.1002/adfm.201907102 PMC10783553

[69] M. B. Oliveira , J. F. Mano , Biotechnol. Prog. 2011, 27, 897. 21584949 10.1002/btpr.618

[70] S. Seiffert , D. A. Weitz , Soft Matter 2010, 6, 3184.

[71] B. G. Chung , K. H. Lee , A. Khademhosseini , S. H. Lee , Lab Chip 2012, 12, 45. 22105780 10.1039/c1lc20859d

[72] Y. Hou , W. Xie , K. Achazi , J. L. Cuellar‐Camacho , M. F. Melzig , W. Chen , R. Haag , Acta Biomater. 2018, 77, 28. 29981495 10.1016/j.actbio.2018.07.003

[73] S. Utech , R. Prodanovic , A. S. Mao , R. Ostafe , D. J. Mooney , D. A. Weitz , Adv. Healthcare Mater. 2015, 4, 1628. 10.1002/adhm.201500021 PMC4529809 26039892

[74] X. Liu , Z. Toprakcioglu , A. J. Dear , A. Levin , F. S. Ruggeri , C. G. Taylor , M. Hu , J. R. Kumita , M. Andreasen , C. M. Dobson , U. Shimanovich , T. P. J. Knowles , Macromol. Rapid Commun. 2019, 40, 1800898. 10.1002/marc.201800898 30840348

[75] S. Guo , T. Yao , X. Ji , C. Zeng , C. Wang , L. Zhang , Angew. Chem., Int. Ed. 2014, 53, 7504. 10.1002/anie.201403256 24898324

[76] Y.‐S. Lin , C.‐H. Yang , Y.‐Y. Hsu , C.‐L. Hsieh , Electrophoresis 2013, 34, 425. 23161405 10.1002/elps.201200282

[77] Y. Tian , E. A. Lipke , ACS Biomater. Sci. Eng. 2020, 6, 6435. 33449645 10.1021/acsbiomaterials.0c00980

[78] J. Wan , Polymers 2012, 4, 1084.

[79] W. J. Duncanson , T. Lin , A. R. Abate , S. Seiffert , R. K. Shah , D. A. Weitz , Lab Chip 2012, 12, 2135. 22510961 10.1039/c2lc21164e

[80] J.‐T. Wang , J. Wang , J.‐J. Han , Small 2011, 7, 1728. 21618428 10.1002/smll.201001913

[81] T. Rossow , P. S. Lienemann , D. J. Mooney , Macromol. Chem. Phys. 2017, 218, 1600380.

[82] D. M. Headen , J. R. García , A. J. García , Microsyst. Nanoeng. 2018, 4, 17076.

[83] M. G. A. Mohamed , S. Kheiri , S. Islam , H. Kumar , A. Yang , K. Kim , Lab Chip 2019, 19, 1621. 30896015 10.1039/c9lc00073a

[84] L. Mazutis , R. Vasiliauskas , D. A. Weitz , Macromol. Biosci. 2015, 15, 1641. 26198619 10.1002/mabi.201500226

[85] D. Liu , H. Zhang , F. Fontana , J. T. Hirvonen , H. A. Santos , Lab Chip 2017, 17, 1856. 28480462 10.1039/c7lc00242d

[86] Z. Lin , J. Wu , W. Qiao , Y. Zhao , K. H. M. Wong , P. K. Chu , L. Bian , S. Wu , Y. Zheng , K. M. C. Cheung , F. Leung , K. W. K. Yeung , Biomaterials 2018, 174, 1. 29763774 10.1016/j.biomaterials.2018.05.011

[87] J. L. Madrigal , S. N. Sharma , K. T. Campbell , R. S. Stilhano , R. Gijsbers , E. A. Silva , Acta Biomater. 2018, 69, 265. 29398644 10.1016/j.actbio.2018.01.013 PMC6819130

[88] S. Tottori , S. Takeuchi , RSC Adv. 2015, 5, 33691.

[89] A. Shima , A. Itou , S. Takeuchi , Sci. Rep. 2020, 10, 288. 31937888 10.1038/s41598-019-57213-0 PMC6959263

[90] S. Sant , D. F. Coutinho , A. K. Gaharwar , N. M. Neves , R. L. Reis , M. E. Gomes , A. Khademhosseini , Adv. Funct. Mater. 2017, 27, 1606273. 31885528 10.1002/adfm.201606273 PMC6934367

[91] A. Patel , Y. Xue , R. Hartley , V. Sant , J. R. Eles , X. T. Cui , D. B. Stolz , S. Sant , Biotechnol. Bioeng. 2018, 115, 2654. 30011077 10.1002/bit.26801 PMC7391220

[92] R. Karnik , F. Gu , P. Basto , C. Cannizzaro , L. Dean , W. Kyei‐Manu , R. Langer , O. C. Farokhzad , Nano Lett. 2008, 8, 2906. 18656990 10.1021/nl801736q

[93] K. Abstiens , A. M. Goepferich , J. Drug Delivery Sci. Technol. 2019, 49, 433.

[94] M. Rhee , P. M. Valencia , M. I. Rodriguez , R. Langer , O. C. Farokhzad , R. Karnik , Adv. Mater. 2011, 23, H79. 21433105 10.1002/adma.201004333 PMC3123733

[95] D. R. Wilson , A. Mosenia , M. P. Suprenant , R. Upadhya , D. Routkevitch , R. A. Meyer , A. Quinones‐Hinojosa , J. J. Green , J. Biomed. Mater. Res., Part A 2017, 105, 1813. 10.1002/jbm.a.36033 28177587

[96] Q. Feng , L. Zhang , C. Liu , X. Li , G. Hu , J. Sun , X. Jiang , Biomicrofluidics 2015, 9, 052604. 26180574 10.1063/1.4922957 PMC4482808

[97] P.‐C. Chen , X. Liu , J. L. Hedrick , Z. Xie , S. Wang , Q.‐Y. Lin , M. C. Hersam , V. P. Dravid , C. A. Mirkin , Science 2016, 352, 1565. 27339985 10.1126/science.aaf8402

[98] R. Rial , P. G. Tahoces , N. Hassan , M. L. Cordero , Z. Liu , J. M. Ruso , Mater. Sci. Eng. C 2019, 102, 221. 10.1016/j.msec.2019.04.037 31146994

[99] N. Hao , Y. Nie , J. X. J. Zhang , Biomater. Sci. 2019, 7, 2218. 30919847 10.1039/c9bm00238c PMC6538461

[100] D. Liu , H. Zhang , S. Cito , J. Fan , E. Mäkilä , J. Salonen , J. Hirvonen , T. M. Sikanen , D. A. Weitz , H. A. Santos , Nano Lett. 2017, 17, 606. 28060521 10.1021/acs.nanolett.6b03251

[101] Y. He , K.‐J. Kim , C.‐H. Chang , Nanotechnology 2017, 28, 235602. 28445169 10.1088/1361-6528/aa6fa7

[102] L. Rao , B. Cai , L. L. Bu , Q. Q. Liao , S. S. Guo , X. Z. Zhao , W. F. Dong , W. Liu , ACS Nano 2017, 11, 3496. 28272874 10.1021/acsnano.7b00133

[103] W. Liu , Y. Zhao , C. Zeng , C. Wang , C. A. Serra , L. Zhang , Chem. Eng. J. 2017, 307, 408.

[104] G. Kibar , U. Çalışkan , E. Y. Erdem , B. Çetin , J. Polym. Sci., Part A‐1: Polym. Chem. 2019, 57, 1396.

[105] X. Yao , G. Zhu , P. Zhu , J. Ma , W. Chen , Z. Liu , T. Kong , Adv. Funct. Mater. 2020, 30, 1909389.

[106] Y. Yu , G. Chen , J. Guo , Y. Liu , J. Ren , T. Kong , Y. Zhao , Mater. Horiz. 2018, 5, 1137.

[107] D. Y. Kim , S. H. Jin , S. G. Jeong , B. Lee , K. K. Kang , C. S. Lee , Sci. Rep. 2018, 8, 8525. 29867182 10.1038/s41598-018-26829-z PMC5986865

[108] Y. Chang , J. Jiang , Wu Chen , W. Yang , L. Chen , P. Chen , J. Shen , S. Qian , T. Zhou , L. Wu , L. Hong , Y. Huang , F. Li , Appl. Mater. Today 2020, 18, 100492. 34746366 10.1016/j.apmt.2019.100492 PMC8570539

[109] R. Rial , R. R. Costa , R. L. Reis , Z. Liu , I. Pashkuleva , J. M. Ruso , Cryst. Growth Des. 2019, 19, 6351.

[110] J. Idaszek , M. Costantini , T. A. Karlsen , J. Jaroszewicz , C. Colosi , S. Testa , E. Fornetti , S. Bernardini , M. Seta , K. Kasarełło , R. Wrzesień , S. Cannata , A. Barbetta , C. Gargioli , J. E. Brinchman , W. Święszkowski , Biofabrication 2019, 11, 044101. 31151123 10.1088/1758-5090/ab2622

[111] L. Serex , A. Bertsch , P. Renaud , Micromachines 2018, 9, 86. 30393362 10.3390/mi9020086 PMC6187762

[112] M. E. Prendergast , J. A. Burdick , Adv. Mater. 2020, 32, 1902516. 10.1002/adma.201902516 31512289

[113] C. Mota , S. Camarero‐Espinosa , M. B. Baker , P. Wieringa , L. Moroni , Chem. Rev. 2020, 120, 10547. 32407108 10.1021/acs.chemrev.9b00789 PMC7564098

[114] C. Colosi , S. R. Shin , V. Manoharan , S. Massa , M. Costantini , A. Barbetta , M. R. Dokmeci , M. Dentini , A. Khademhosseini , Adv. Mater. 2016, 28, 677. 26606883 10.1002/adma.201503310 PMC4804470

[115] S. Derakhshanfar , R. Mbeleck , K. Xu , X. Zhang , W. Zhong , M. Xing , Bioact. Mater. 2018, 3, 144. 29744452 10.1016/j.bioactmat.2017.11.008 PMC5935777

[116] M. Costantini , J. Jaroszewicz , Ł. Kozoń , K. Szlązak , W. Święszkowski , P. Garstecki , C. Stubenrauch , A. Barbetta , J. Guzowski , Angew. Chem., Int. Ed. 2019, 58, 7620. 10.1002/anie.201900530 30908850

[117] A. K. Miri , D. Nieto , L. Iglesias , H. Goodarzi Hosseinabadi , S. Maharjan , G. Ulises Ruiz‐Esparza , P. Khoshakhlagh , A. Manbachi , Adv. Mater. 2018, 30, 1800242. 10.1002/adma.201800242 PMC6133710 29737048

[118] A. M. Leferink , D. Schipper , E. Arts , E. Vrij , N. Rivron , M. Karperien , K. Mittmann , C. Van Blitterswijk , L. Moroni , R. Truckenmüller , Adv. Mater. 2014, 26, 2592. 24395427 10.1002/adma.201304539

[119] A. M. Leferink , M. P. Tibbe , E. G. B. M. Bossink , L. E. de Heus , H. van Vossen , A. van den Berg , L. Moroni , R. K. Truckenmüller , Mater. Today Bio 2019, 4, 100025. 10.1016/j.mtbio.2019.100025 PMC7061620 32159154

[120] J. Xu , D. H. C. Wong , J. D. Byrne , K. Chen , C. Bowerman , J. M. Desimone , Angew. Chem., Int. Ed. 2013, 52, 6580. 10.1002/anie.201209145 PMC4157646 23670869

[121] M. Caldorera‐Moore , M. K. Kang , Z. Moore , V. Singh , S. V. Sreenivasan , L. Shi , R. Huang , K. Roy , Soft Matter 2011, 7, 2879.

[122] H. Tekin , T. Tsinman , J. G. Sanchez , B. J. Jones , G. Camci‐Unal , J. W. Nichol , R. Langer , A. Khademhosseini , J. Am. Chem. Soc. 2011, 133, 12944. 21766872 10.1021/ja204266a PMC3206098

[123] F. Fontana , J. P. Martins , G. Torrieri , H. A. Santos , Adv. Mater. Technol. 2019, 4, 1970034.

[124] G. C. Le Goff , J. Lee , A. Gupta , W. A. Hill , P. S. Doyle , Adv. Sci. 2015, 2, 1500149. 10.1002/advs.201500149 PMC5115321 27980910

[125] Y. Yi , L. Sanchez , Y. Gao , Y. Yu , Analyst 2016, 141, 3526. 27052001 10.1039/c6an00325g PMC4899188

[126] H. J. M. Wolff , J. Linkhorst , T. Göttlich , J. Savinsky , A. J. D. Krüger , L. De Laporte , M. Wessling , Lab Chip 2020, 20, 285. 31802080 10.1039/c9lc00749k

[127] B. Xue , V. Kozlovskaya , E. Kharlampieva , J. Mater. Chem. B 2017, 5, 9. 32263432 10.1039/c6tb02746f

[128] D. Barata , E. Provaggi , C. Van Blitterswijk , P. Habibovic , Lab Chip 2017, 17, 4134. 29114689 10.1039/c7lc00802c

[129] M. Nouri‐Goushki , A. Sharma , L. Sasso , S. Zhang , B. C. J. Van Der Eerden , U. Staufer , L. E. Fratila‐Apachitei , A. A. Zadpoor , ACS Biomater. Sci. Eng. 2019, 5, 6127. 33405666 10.1021/acsbiomaterials.9b01155

[130] F. Ye , J. Jiang , H. Chang , L. Xie , J. Deng , Z. Ma , W. Yuan , Biomicrofluidics 2015, 9, 044106. 26339307 10.1063/1.4926807 PMC4514715

[131] D. Barata , A. Resmini , D. Pereira , S. A. Veldhuis , C. A. van Blitterswijk , J. E. ten Elshof , P. Habibovic , J. Mater. Chem. B 2016, 4, 1044. 32262996 10.1039/c5tb02027a

[132] E. Müller , T. Pompe , U. Freudenberg , C. Werner , Adv. Mater. 2017, 29, 1703489. 10.1002/adma.201703489 28960524

[133] L. Zhang , S. Chen , R. Liang , Yi Chen , S. Li , S. Li , Z. Sun , Y. Wang , G. Li , A. Ming , Y. Yang , J. Biomed. Mater. Res., Part A 2018, 106, 3123. 10.1002/jbm.a.36507 30260557

[134] A. Nandakumar , R. K. Truckenmüller , M. Ahmed , F. Damanik , D. R. Santos , N. Auffermann , J. de Boer , P. Habibovic , C. van Blitterswijk , L. Moroni , Small 2013, 9, 3405. 23447336 10.1002/smll.201300220

[135] S. Liang , J. Li , X. Li , J. Man , J. K. Nunes , H. Chen , J. Am. Ceram. Soc. 2018, 101, 3787.

[136] L. Yi , J. Liu , Int. Mater. Rev. 2017, 62, 415.

[137] L. Zhu , B. Wang , S. Handschuh‐Wang , X. Zhou , Small 2020, 16, 1903841. 10.1002/smll.201903841 31573755

[138] K. Khoshmanesh , S. Y. Tang , J. Y. Zhu , S. Schaefer , A. Mitchell , K. Kalantar‐Zadeh , M. D. Dickey , Lab Chip 2017, 17, 974. 28225135 10.1039/c7lc00046d

[139] B. Zhu , Y. Cai , Z. Wu , F. Niu , H. Yang , IEEE Access 2019, 7, 152224.

[140] N. Hallfors , A. Khan , M. D. Dickey , A. M. Taylor , Lab Chip 2013, 13, 522. 23232866 10.1039/c2lc40954b PMC4394010

[141] W. Su , S. A. Nauroze , B. Ryan , M. M. Tentzeris , in IEEE MTT‐S Int. Microwave Symp. (IMS), IEEE, Piscataway, NJ 2017, pp. 1579–1582.

[142] D. Barata , C. Van Blitterswijk , P. Habibovic , Acta Biomater. 2016, 34, 1. 26361719 10.1016/j.actbio.2015.09.009

[143] P. M. Valencia , E. M. Pridgen , M. Rhee , R. Langer , O. C. Farokhzad , R. Karnik , ACS Nano 2013, 7, 10671. 24215426 10.1021/nn403370e PMC3963607

[144] H. F. Chan , S. Ma , K. W. Leong , Regener. Biomater. 2016, 3, 87. 10.1093/rb/rbw009 PMC4817324 27047674

[145] M. C. Operti , D. Fecher , E. A. W. van Dinther , S. Grimm , R. Jaber , C. G. Figdor , O. Tagit , Int. J. Pharm. 2018, 550, 140. 30144511 10.1016/j.ijpharm.2018.08.044

[146] E. M. Benetti , M. K. Gunnewiek , C. A. Van Blitterswijk , G. J. Vancso , L. Moroni , J. Mater. Chem. B 2016, 4, 4244. 32263405 10.1039/c6tb00947f

[147] G. Mestres , R. A. Perez , N. L. D'Elía , L. Barbe , Biomed. Phys. Eng. Express 2019, 5, 032001.

[148] S. Siddiqui , A. Chandrasekaran , N. Lin , N. Tufenkji , C. Moraes , Langmuir 2019, 35, 8840. 31177781 10.1021/acs.langmuir.9b00803

[149] A. Hartmann , M. Stamp , R. Kmeth , S. Buchegger , B. Stritzker , B. Saldamli , R. Burgkart , M. F. Schneider , A. Wixforth , Lab Chip 2014, 14, 542. 24292668 10.1039/c3lc50916h

[150] M. Stamp , A. Jötten , P. Kudella , D. Breyer , F. Strobl , T. Geislinger , A. Wixforth , C. Westerhausen , Diagnostics 2016, 6, 38. 27775638 10.3390/diagnostics6040038 PMC5192513

[151] C. M. Dumont , P. Karande , D. M. Thompson , Tissue Eng., Part C 2014, 20, 620. 10.1089/ten.tec.2013.0362 PMC4115783 24256302

[152] S. Naskar , A. K. Panda , V. Kumaran , B. Mehta , B. Basu , ACS Appl. Bio Mater. 2018, 1, 414. 10.1021/acsabm.8b00147 35016400

[153] S. Kheiri , M. G. A. Mohamed , M. Amereh , D. Roberts , K. Kim , Mater. Sci. Eng. C 2020, 111, 110754. 10.1016/j.msec.2020.110754 32279821

[154] W. Tong , X. Yao , S. Duan , B. Yu , X. Ding , X. Ding , F.‐J. Xu , Langmuir 2020, 36, 354. 31826611 10.1021/acs.langmuir.9b02747

[155] C. H. Ma , H. B. Zhang , S. M. Yang , R. X. Yin , X. J. Yao , W. J. Zhang , Biomicrofluidics 2018, 12, 034106. 29861809 10.1063/1.5021394 PMC5959737

[156] S. A. Castleberry , W. Li , D. Deng , S. Mayner , P. T. Hammond , ACS Nano 2014, 8, 6580. 24836460 10.1021/nn501963q PMC4133994

[157] J. He , Y. Du , Y. Guo , M. J. Hancock , B. Wang , H. Shin , J. Wu , D. Li , A. Khademhosseini , Biotechnol. Bioeng. 2011, 108, 175. 20721897 10.1002/bit.22901 PMC3013224

[158] J. Dou , S. Mao , H. Li , J.‐M. Lin , Anal. Chem. 2020, 92, 892. 31790197 10.1021/acs.analchem.9b03681

[159] Y. Kim , P. Baipaywad , Y. Jeong , H. Park , Int. J. Biol. Macromol. 2018, 110, 472. 29369781 10.1016/j.ijbiomac.2018.01.046

[160] N. R. Labriola , E. Mathiowitz , E. M. Darling , Biomater. Sci. 2017, 41. 10.1039/c6bm00692b PMC5201106 27935612

[161] T. Anada , T. Sato , T. Kamoya , Y. Shiwaku , K. Tsuchiya , T. Takano‐Yamamoto , K. Sasaki , O. Suzuki , Regener. Ther. 2016, 3, 58. 10.1016/j.reth.2016.02.004 PMC6581819 31245473

[162] S. Sart , R. F. X. Tomasi , G. Amselem , C. N. Baroud , Nat. Commun. 2017, 8, 469. 28883466 10.1038/s41467-017-00475-x PMC5589863

[163] H. V. Unadkat , M. Hulsman , K. Cornelissen , B. J. Papenburg , R. K. Truckenmuller , A. E. Carpenter , M. Wessling , G. F. Post , M. Uetz , M. J. T. Reinders , D. Stamatialis , C. A. Van Blitterswijk , J. De Boer , Proc. Natl. Acad. Sci. USA 2011, 108, 16565. 21949368 10.1073/pnas.1109861108 PMC3189082

[164] Y. Zhao , R. K. Truckenmüller , M. Levers , W. S. Hua , J. de Boer , B. Papenburg , Mater. Sci. Eng. C 2017, 71, 558. 10.1016/j.msec.2016.11.004 27987744

[165] A. A. K. Moe , M. Suryana , G. Marcy , S. K. Lim , S. Ankam , J. Z. W. Goh , J. Jin , B. K. K. Teo , J. B. K. Law , H. Y. Low , E. L. K. Goh , M. P. Sheetz , E. K. F. Yim , Small 2012, 8, 3050. 22807278 10.1002/smll.201200490

[166] N. R. M. Beijer , A. S. Vasilevich , B. Pilavci , R. K. Truckenmüller , Y. Zhao , S. Singh , B. J. Papenburg , J. de Boer , Adv. Biosyst. 2017, 1, 1700002. 10.1002/adbi.201700002 32646161

[167] F. F. B. Hulshof , Y. Zhao , A. Vasilevich , N. R. M. Beijer , M. de Boer , B. J. Papenburg , C. van Blitterswijk , D. Stamatialis , J. de Boer , Acta Biomater. 2017, 62, 188. 28823718 10.1016/j.actbio.2017.08.023

[168] L. Burroughs , M. H. Amer , M. Vassey , B. Koch , G. P. Figueredo , B. Mukonoweshuro , P. Mikulskis , A. Vasilevich , S. Vermeulen , I. L. Dryden , D. A. Winkler , A. M. Ghaemmaghami , F. R. A. J. Rose , J. De Boer , M. R. Alexander , Biomaterials 2021, 271, 120740. 33714019 10.1016/j.biomaterials.2021.120740

[169] A. C. G. Weiss , K. Kempe , S. Förster , F. Caruso , Biomacromolecules 2018, 19, 2580. 29668268 10.1021/acs.biomac.8b00196

[170] A. C. G. Weiss , K. Krüger , Q. A. Besford , M. Schlenk , K. Kempe , S. Förster , F. Caruso , ACS Appl. Mater. Interfaces 2019, 11, 2459. 30600987 10.1021/acsami.8b14307

[171] D. Pozzi , G. Caracciolo , L. Digiacomo , V. Colapicchioni , S. Palchetti , A. L. Capriotti , C. Cavaliere , R. Zenezini Chiozzi , A. Puglisi , A. Laganà , Nanoscale 2015, 7, 13958. 26222625 10.1039/c5nr03701h

[172] M. Björnmalm , M. Faria , F. Caruso , J. Am. Chem. Soc. 2016, 138, 13449. 27672703 10.1021/jacs.6b08673

[173] L. Liu , Y. Koo , B. Collins , Z. Xu , J. Sankar , Y. Yun , PLoS One 2017, 12, e0182914. 28797069 10.1371/journal.pone.0182914 PMC5552284

[174] S. Mao , Q. Zhang , H. Li , Q. Huang , M. Khan , K. Uchiyama , J.‐M. Lin , Anal. Chem. 2018, 90, 9637. 30016872 10.1021/acs.analchem.8b02653

[175] A. C. Ceccacci , C.‐H. Chen , E.‐T. Hwu , L. Morelli , S. Bose , F. G. Bosco , S. Schmid , A. Boisen , Sens. Actuators, B 2017, 241, 1303.

[176] T. Kumeria , K. Gulati , A. Santos , D. Losic , ACS Appl. Mater. Interfaces 2013, 5, 5436. 23731441 10.1021/am4013984

[177] Z. Li , E. Seker , Lab Chip 2017, 17, 3331. 28868535 10.1039/c7lc00851a

[178] J. Ren , J. Li , Y. Li , P. Xiao , Y. Liu , C. M. Tsang , S. W. Tsao , D. Lau , K. W. Y. Chan , R. H. W. Lam , ACS Biomater. Sci. Eng. 2019, 5, 3889. 33438428 10.1021/acsbiomaterials.8b01273

[179] Y. Niu , X. Zhang , T. Si , Y. Zhang , L. Qi , G. Zhao , R. X. Xu , X. He , Y. Zhao , Small 2017, 13, 1702821. 10.1002/smll.201702821 29140604

[180] E. R. Castro , M. D. Tarn , P. Ginterová , H. Zhu , Y. Xu , P. Neužil , Microfluid. Nanofluid. 2018, 22, 51.

[181] X. Feng , B.‐F. Liu , J. Li , X. Liu , Mass Spectrom. Rev. 2015, 34, 535. 24399782 10.1002/mas.21417

[182] C. Yu , F. Tang , X. Qian , Y. Chen , Q. Yu , K. Ni , X. Wang , Sci. Rep. 2017, 7, 17389. 29234133 10.1038/s41598-017-17764-6 PMC5727197

[183] Y. Zhao , M. Tang , F. Liu , H. Li , H. Wang , D. Xu , Anal. Chem. 2019, 91, 13418. 31566960 10.1021/acs.analchem.9b01844

[184] D.‐S. Wang , S.‐K. Fan , Sensors 2016, 16, 1175.27472340

[185] Y. Song , B. Lin , T. Tian , X. Xu , W. Wang , Q. Ruan , J. Guo , Z. Zhu , C. Yang , Anal. Chem. 2019, 91, 388. 30412383 10.1021/acs.analchem.8b05007

[186] A. Guan , P. Hamilton , Y. Wang , M. Gorbet , Z. Li , K. S. Phillips , Nat. Biomed. Eng 2017, 1, 0045.

[187] Y. Wang , A. Guan , S. Wickramasekara , K. S. Phillips , Annu. Rev. Anal. Chem. 2018, 11, 307. 10.1146/annurev-anchem-061417-125556 29579404

[188] D. Arora , G. Babakhanova , C. G. Simon , ACS Biomater. Sci. Eng. 2020, 6, 5368. 33320558 10.1021/acsbiomaterials.0c00475

[189] C. S. Hughes , L. M. Postovit , G. A. Lajoie , Proteomics 2010, 10, 1886. 20162561 10.1002/pmic.200900758

[190] H. Geckil , F. Xu , X. Zhang , S. Moon , U. Demirci , Nanomedicine 2010, 5, 469. 20394538 10.2217/nnm.10.12 PMC2892416

[191] N. Broguiere , L. Isenmann , C. Hirt , T. Ringel , S. Placzek , E. Cavalli , F. Ringnalda , L. Villiger , R. Züllig , R. Lehmann , G. Rogler , M. H. Heim , J. Schüler , M. Zenobi‐Wong , G. Schwank , Adv. Mater. 2018, 30, 1801621. 10.1002/adma.201801621 30203567

[192] R. L. Wilson , G. Swaminathan , K. Ettayebi , C. Bomidi , X. L. Zeng , S. E. Blutt , M. K. Estes , K. J. Grande‐Allen , Tissue Eng., Part C 2021, 27, 12. 10.1089/ten.tec.2020.0306 PMC7826425 33334213

[193] N. Jusoh , S. Oh , S. Kim , J. Kim , N. L. Jeon , Lab Chip 2015, 15, 3984. 26288174 10.1039/c5lc00698h

[194] Q. Sun , S. Choudhary , C. Mannion , Y. Kissin , J. Zilberberg , W. Y. Lee , Bone 2018, 106, 148. 29066313 10.1016/j.bone.2017.10.019 PMC5694355

[195] A. Tangprasert , C. Tansakul , N. Thuaksubun , J. Meesane , Mater. Des. 2017, 134, 486.

[196] D. M. Wuest , A. M. Wing , K. H. Lee , J. Neurosci. Methods 2013, 212, 211. 23131353 10.1016/j.jneumeth.2012.10.016

[197] X. Shao , D. Gao , Y. Chen , F. Jin , G. Hu , Y. Jiang , H. Liu , Anal. Chim. Acta 2016, 934, 186. 27506359 10.1016/j.aca.2016.06.028

[198] D. Kim , S. Eom , S. M. Park , H. Hong , D. S. Kim , Sci. Rep. 2019, 9, 14915. 31624315 10.1038/s41598-019-51560-8 PMC6797789

[199] J. Yoo , T. H. Kim , S. Park , K. Char , S. H. Kim , J. J. Chung , Y. Jung , Adv. Funct. Mater. 2021, 31, 2008172.

[200] T. Pasman , D. Grijpma , D. Stamatialis , A. Poot , J. R. Soc., Interface 2018, 15, 20180351. 30045892 10.1098/rsif.2018.0351 PMC6073644

[201] D. Huh , B. D. Matthews , A. Mammoto , M. Montoya‐Zavala , H. Y. Hsin , D. E. Ingber , Science 2010, 328, 1662. 20576885 10.1126/science.1188302 PMC8335790

[202] H. J. Kim , D. Huh , G. Hamilton , D. E. Ingber , Lab Chip 2012, 12, 2165. 22434367 10.1039/c2lc40074j

[203] D. Huh , Ann. Am. Thorac. Soc. 2015, 12, S42. 25830834 10.1513/AnnalsATS.201410-442MG PMC5467107

[204] J. W. Lee , J. S. Sung , Y. S. Park , S. Chung , Y. H. Kim , BioTechniques 2018, 65, 197. 30284938 10.2144/btn-2018-0046

[205] E. Vrij , J. Rouwkema , V. Lapointe , C. Van Blitterswijk , R. Truckenmüller , N. Rivron , Adv. Mater. 2016, 28, 4032. 27000493 10.1002/adma.201505723

[206] P. Kakni , R. Hueber , K. Knoops , C. López‐Iglesias , R. Truckenmüller , P. Habibovic , S. Giselbrecht , Adv. Biosyst. 2020, 4, 2000126. 10.1002/adbi.202000126 32734713

[207] M. Cabodi , N. W. Choi , J. P. Gleghorn , C. S. D. Lee , L. J. Bonassar , A. D. Stroock , J. Am. Chem. Soc. 2005, 127, 13788. 16201789 10.1021/ja054820t

[208] J. A. Jiménez‐Torres , S. L. Peery , K. E. Sung , D. J. Beebe , Adv. Healthcare Mater. 2016, 5, 198. 10.1002/adhm.201500608 PMC4776323 26610188

[209] M. T. Lam , Y. C. Huang , R. K. Birla , S. Takayama , Biomaterials 2009, 30, 1150. 19064284 10.1016/j.biomaterials.2008.11.014

[210] G. Kaushik , J. Leijten , A. Khademhosseini , Stem Cells 2017, 35, 51. 27641724 10.1002/stem.2502 PMC6527109

[211] S. Ahadian , R. Civitarese , D. Bannerman , M. H. Mohammadi , R. Lu , E. Wang , L. Davenport‐Huyer , B. Lai , B. Zhang , Y. Zhao , S. Mandla , A. Korolj , M. Radisic , Adv. Healthcare Mater. 2018, 7, 1700506. 10.1002/adhm.201800734 30134074

[212] D. Mandt , P. Gruber , M. Markovic , M. Tromayer , M. Rothbauer , S. R. A. Krayz , F. Ali , J. Van Hoorick , W. Holnthoner , S. Mühleder , P. Dubruel , S. Van Vlierberghe , P. Ertl , R. Liska , A. Ovsianikov , Int. J. Bioprint. 2018, 4, 144. 33102920 10.18063/IJB.v4i2.144 PMC7581993

[213] H. Lee , D. W. Cho , Lab Chip 2016, 16, 2618. 27302471 10.1039/c6lc00450d

[214] I. Maschmeyer , T. Hasenberg , A. Jaenicke , M. Lindner , A. K. Lorenz , J. Zech , L. A. Garbe , F. Sonntag , P. Hayden , S. Ayehunie , R. Lauster , U. Marx , E. M. Materne , Eur. J. Pharm. Biopharm. 2015, 95, 77. 25857839 10.1016/j.ejpb.2015.03.002 PMC6574126

[215] P. Occhetta , A. Mainardi , E. Votta , Q. Vallmajo‐Martin , M. Ehrbar , I. Martin , A. Barbero , M. Rasponi , Nat. Biomed. Eng. 2019, 3, 545. 31160722 10.1038/s41551-019-0406-3

[216] M. B. Esch , H. Ueno , D. R. Applegate , M. L. Shuler , Lab Chip 2016, 16, 2719. 27332143 10.1039/c6lc00461j

[217] N. Santhanam , L. Kumanchik , X. Guo , F. Sommerhage , Y. Cai , M. Jackson , C. Martin , G. Saad , C. W. Mcaleer , Y. Wang , A. Lavado , C. J. Long , J. J. Hickman , Biomaterials 2018, 166, 64. 29547745 10.1016/j.biomaterials.2018.02.047 PMC5866791

[218] C. D. Edington , W. L. K. Chen , E. Geishecker , T. Kassis , L. R. Soenksen , B. M. Bhushan , D. Freake , J. Kirschner , C. Maass , N. Tsamandouras , J. Valdez , C. D. Cook , T. Parent , S. Snyder , J. Yu , E. Suter , M. Shockley , J. Velazquez , J. J. Velazquez , L. Stockdale , J. P. Papps , I. Lee , N. Vann , M. Gamboa , M. E. Labarge , Z. Zhong , X. Wang , L. A. Boyer , D. A. Lauffenburger , R. L. Carrier , C. Communal , S. R. Tannenbaum , C. L. Stokes , D. J. Hughes , G. Rohatgi , D. L. Trumper , M. Cirit , L. G. Griffith , Sci. Rep. 2018, 8, 4530. 29540740 10.1038/s41598-018-22749-0 PMC5852083

[219] C. Oleaga , C. Bernabini , A. S. T. Smith , B. Srinivasan , M. Jackson , W. Mclamb , V. Platt , R. Bridges , Y. Cai , N. Santhanam , B. Berry , S. Najjar , N. Akanda , X. Guo , C. Martin , G. Ekman , M. B. Esch , J. Langer , G. Ouedraogo , J. Cotovio , L. Breton , M. L. Shuler , J. J. Hickman , Sci. Rep. 2016, 6, 20030. 26837601 10.1038/srep20030 PMC4738272

[220] A. Skardal , S. V. Murphy , M. Devarasetty , I. Mead , H. W. Kang , Y. J. Seol , Yu Shrike Zhang , Su‐R Shin , L. Zhao , J. Aleman , A. R. Hall , T. D. Shupe , A. Kleensang , M. R. Dokmeci , S. Jin Lee , J. D. Jackson , J. J. Yoo , T. Hartung , A. Khademhosseini , S. Soker , C. E. Bishop , A. Atala , Sci. Rep. 2017, 7, 8837. 28821762 10.1038/s41598-017-08879-x PMC5562747

[221] G. Sorrentino , S. Rezakhani , E. Yildiz , S. Nuciforo , M. H. Heim , M. P. Lutolf , K. Schoonjans , Nat. Commun. 2020, 11, 3416. 32651372 10.1038/s41467-020-17161-0 PMC7351772

[222] M. M. Capeling , M. Czerwinski , S. Huang , Yu‐H Tsai , A. Wu , M. S. Nagy , B. Juliar , N. Sundaram , Y. Song , W. M. Han , S. Takayama , E. Alsberg , A. J. Garcia , M. Helmrath , A. J. Putnam , J. R. Spence , Stem Cell Rep. 2019, 12, 381. 10.1016/j.stemcr.2018.12.001 PMC6373433 30612954

[223] R. Cruz‐Acuña , M. Quirós , A. E. Farkas , P. H. Dedhia , S. Huang , D. Siuda , V. García‐Hernández , A. J. Miller , J. R. Spence , A. Nusrat , A. J. García , Nat. Cell Biol. 2017, 19, 1326. 29058719 10.1038/ncb3632 PMC5664213

[224] N. S. Bhise , V. Manoharan , S. Massa , A. Tamayol , M. Ghaderi , M. Miscuglio , Qi Lang , Yu Shrike Zhang , Su R Shin , G. Calzone , N. Annabi , T. D. Shupe , C. E. Bishop , A. Atala , M. R. Dokmeci , A. Khademhosseini , Biofabrication 2016, 8, 014101. 26756674 10.1088/1758-5090/8/1/014101

[225] S. L. Natividad‐Diaz , S. Browne , A. K. Jha , Z. Ma , S. Hossainy , Y. K. Kurokawa , S. C. George , K. E. Healy , Biomaterials 2019, 194, 73. 30583150 10.1016/j.biomaterials.2018.11.032 PMC6453535

[226] K. M. Lugo‐Cintrón , J. M. Ayuso , B. R. White , P. M. Harari , S. M. Ponik , D. J. Beebe , M. M. Gong , M. Virumbrales‐Muñoz , Lab Chip 2020, 10. 10.1039/d0lc00099j PMC7330815 32297896

[227] S. Hao , L. Ha , G. Cheng , Y. Wan , Y. Xia , D. M. Sosnoski , A. M. Mastro , S. Y. Zheng , Small 2018, 14, 1702787. 10.1002/smll.201702787 29399951

[228] H. Mollica , R. Palomba , R. Primavera , P. Decuzzi , ACS Biomater. Sci. Eng. 2019, 5, 4834. 33448826 10.1021/acsbiomaterials.9b00697

[229] R. Zhang , N. B. Larsen , Lab Chip 2017, 17, 4273. 29116271 10.1039/c7lc00926g

[230] L. E. Bertassoni , M. Cecconi , V. Manoharan , M. Nikkhah , J. Hjortnaes , A. L. Cristino , G. Barabaschi , D. Demarchi , M. R. Dokmeci , Y. Yang , A. Khademhosseini , Lab Chip 2014, 14, 2202. 24860845 10.1039/c4lc00030g PMC4201051

[231] M. Tenje , F. Cantoni , A. M. Porras Hernández , S. S. Searle , S. Johansson , L. Barbe , M. Antfolk , H. Pohlit , Organs‐on‐a‐Chip 2020, 2, 100003.

[232] E. Rosella , N. Jia , D. Mantovani , J. Greener , J. Mater. Sci. Technol. 2021, 63, 54.

[233] J. S. Lee , R. Romero , Y. M. Han , H. C. Kim , C. J. Kim , J.‐S. Hong , D. Huh , J. Matern.‐Fetal Neonat. Med. 2016, 29, 1046. 10.3109/14767058.2015.1038518 PMC5625348 26075842

[234] C. Wang , N. Tanataweethum , S. Karnik , A. Bhushan , ACS Biomater. Sci. Eng. 2018, 4, 1377. 33418668 10.1021/acsbiomaterials.7b00883

[235] V. V. Abhyankar , M. Wu , C. Koh , A. V. Hatch , PLoS One 2016, 11, e0156341. 27227828 10.1371/journal.pone.0156341 PMC4881956

[236] P. Das , A. D. Van Der Meer , A. Vivas , Y. B. Arik , J. C. Remigy , J. F. Lahitte , R. G. H. Lammertink , P. Bacchin , Tissue Eng., Part A 2019, 25, 1635. 30957672 10.1089/ten.TEA.2019.0021

[237] N. Jusoh , S. Oh , S. Kim , J. Kim , N. L. Jeon , Lab Chip 2015, 15, 3984. 26288174 10.1039/c5lc00698h

[238] E. Budyn , N. Gaci , S. Sanders , M. Bensidhoum , E. Schmidt , B. Cinquin , P. Tauc , H. Petite , MRS Adv. 2018, 3, 1443.

[239] A. Marturano‐Kruik , M. M. Nava , K. Yeager , A. Chramiec , L. Hao , S. Robinson , E. Guo , M. T. Raimondi , G. Vunjak‐Novakovic , Proc. Natl. Acad. Sci. USA 2018, 115, 1256. 29363599 10.1073/pnas.1714282115 PMC5819403

[240] D. Huh , H. J. Kim , J. P. Fraser , D. E. Shea , M. Khan , A. Bahinski , G. A. Hamilton , D. E. Ingber , Nat. Protoc. 2013, 8, 2135. 24113786 10.1038/nprot.2013.137

[241] M. W. Toepke , D. J. Beebe , Lab Chip 2006, 6, 1484. 17203151 10.1039/b612140c

[242] B. M. Maoz , A. Herland , O. Y. F. Henry , W. D. Leineweber , M. Yadid , J. Doyle , R. Mannix , V. J. Kujala , E. A. Fitzgerald , K. K. Parker , D. E. Ingber , Lab Chip 2017, 17, 2294. 28608907 10.1039/c7lc00412e

[243] H. J. Kim , H. Li , J. J. Collins , D. E. Ingber , Proc. Natl. Acad. Sci. USA 2016, 113, E7. 26668389 10.1073/pnas.1522193112 PMC4711860

[244] F. Sorba , A. Poulin , R. Ischer , H. Shea , C. Martin‐Olmos , Lab Chip 2019, 19, 2138. 31115420 10.1039/c9lc00075e

[245] J. M. Lee , D. Y. Park , L. Yang , E. J. Kim , C. D. Ahrberg , K. B. Lee , B. G. Chung , Sci. Rep. 2018, 8, 17145. 30464248 10.1038/s41598-018-35216-7 PMC6249215

[246] E. J. Vrij , S. Espinoza , M. Heilig , A. Kolew , M. Schneider , C. A. van Blitterswijk , R. K. Truckenmüller , N. C. Rivron , Lab Chip 2016, 16, 734. 26775648 10.1039/c5lc01499a

[247] P. Samal , P. Maurer , C. Blitterswijk , R. K. Truckenmüller , S. Giselbrecht , Adv. Mater. 2020, 32, 1907966. 10.1002/adma.201907966 32346909

[248] M. Buitinga , R. K. Truckenmüller , M. A. Engelse , L. Moroni , H. W. M. Ten Hoopen , C. A. van Blitterswijk , E. J. de Koning , A. A. van Apeldoorn , M. Karperien , PLoS One 2013, 8, e64772. 23737999 10.1371/journal.pone.0064772 PMC3667808

[249] M. Buitinga , F. Assen , M. Hanegraaf , P. Wieringa , J. Hilderink , L. Moroni , R. Truckenmüller , C. Van Blitterswijk , G. W. Römer , F. Carlotti , E. De Koning , M. Karperien , A. Van Apeldoorn , Biomaterials 2017, 135, 10. 28478326 10.1016/j.biomaterials.2017.03.031

[250] R. K. Truckenmüller , S. Giselbrecht , M. Escalante‐Marun , M. Groenendijk , B. Papenburg , N. Rivron , H. Unadkat , V. Saile , V. Subram , R. K. Truckenmüller , S. Giselbrecht , M. Escalante‐Marun , M. Groenendijk , B. Papenburg , N. Rivron , H. Unadkat , V. Saile , V. Subramaniam , A. Van Den Berg , C. van Blitterswijk , M. Wessling , J. de Boer , D. Stamatialis , Biomed. Microdevices 2012, 14, 95. 22048776 10.1007/s10544-011-9588-5 PMC3288368

[251] K. Gold , A. K. Gaharwar , A. Jain , Biomaterials 2019, 196, 2. 30072038 10.1016/j.biomaterials.2018.07.029 PMC6344330

[252] A. Malheiro , P. Wieringa , C. Mota , M. Baker , L. Moroni , ACS Biomater. Sci. Eng. 2016, 2, 1694. 33440469 10.1021/acsbiomaterials.6b00269

[253] M. L. Rathod , J. Ahn , N. L. Jeon , J. Lee , Lab Chip 2017, 17, 2508. 28653725 10.1039/c7lc00340d

[254] P. N. Ingram , L. E. Hind , J. A. Jiminez‐Torres , A. Huttenlocher , D. J. Beebe , Adv. Healthcare Mater. 2018, 7, 1700497. 10.1002/adhm.201700497 PMC6402823 29364596

[255] J. A. Jiménez‐Torres , M. Virumbrales‐Muñoz , K. E. Sung , M. H. Lee , E. J. Abel , D. J. Beebe , EBioMedicine 2019, 42, 408. 30902740 10.1016/j.ebiom.2019.03.026 PMC6491391

[256] M. M. Morgan , M. K. Livingston , J. W. Warrick , E. M. Stanek , E. T. Alarid , D. J. Beebe , B. P. Johnson , Sci. Rep. 2018, 8, 7139. 29740030 10.1038/s41598-018-25461-1 PMC5940820

[257] A. C. Daly , P. Pitacco , J. Nulty , G. M. Cunniffe , D. J. Kelly , Biomaterials 2018, 162, 34. 29432987 10.1016/j.biomaterials.2018.01.057

[258] S. Zhao , Y. Chen , B. P. Partlow , A. S. Golding , P. Tseng , J. Coburn , M. B. Applegate , J. E. Moreau , F. G. Omenetto , D. L. Kaplan , Biomaterials 2016, 93, 60. 27077566 10.1016/j.biomaterials.2016.03.041

[259] K. A. Heintz , M. E. Bregenzer , J. L. Mantle , K. H. Lee , J. L. West , J. H. Slater , Adv. Healthcare Mater. 2016, 5, 2153. 10.1002/adhm.201600351 PMC5014628 27239785

[260] T.‐C. Tseng , F.‐Y. Hsieh , P. Theato , Y. Wei , S. Hsu , Biomaterials 2017, 133, 20. 28414976 10.1016/j.biomaterials.2017.04.008

[261] H. W. Kang , S. J. Lee , I. K. Ko , C. Kengla , J. J. Yoo , A. Atala , Nat. Biotechnol. 2016, 34, 312. 26878319 10.1038/nbt.3413

[262] H. Takahashi , T. Okano , Adv. Healthcare Mater. 2015, 4, 2388. 10.1002/adhm.201500194 26033874

[263] O. Guillaume‐Gentil , M. Gabi , M. Zenobi‐Wong , J. Vörös , Biomed. Microdevices 2011, 13, 221. 21057978 10.1007/s10544-010-9487-1

[264] O. Guillaume‐Gentil , O. V. Semenov , A. H. Zisch , R. Zimmermann , J. Vörös , M. Ehrbar , Biomaterials 2011, 32, 4376. 21458856 10.1016/j.biomaterials.2011.02.058

[265] B. Zhang , M. Montgomery , M. D. Chamberlain , S. Ogawa , A. Korolj , A. Pahnke , L. A. Wells , S. Massé , J. Kim , L. Reis , A. Momen , S. S. Nunes , A. R. Wheeler , K. Nanthakumar , G. Keller , M. V. Sefton , M. Radisic , Nat. Mater. 2016, 15, 669. 26950595 10.1038/nmat4570 PMC4879054

[266] M. P. Tibbe , A. M. Leferink , A. van den Berg , J. C. T. Eijkel , L. I. Segerink , Adv. Mater. Technol. 2018, 3, 1700200.

[267] G. A. Higuera , J. A. A. Hendriks , J. Van Dalum , L. Wu , R. Schotel , L. Moreira‐Teixeira , M. Van Den Doel , J. C. H. Leijten , J. Riesle , M. Karperien , C. A. Van Blitterswijk , L. Moroni , Integr. Biol. 2013, 5, 889. 10.1039/c3ib40023a 23652478

[268] C. M. França , A. Tahayeri , N. S. Rodrigues , S. Ferdosian , R. M. Puppin Rontani , G. Sereda , J. L. Ferracane , L. E. Bertassoni , Lab Chip 2020, 20, 405. 31854401 10.1039/c9lc00915a PMC7395925

[269] M. A. J. Morsink , N. G. A. Willemen , J. Leijten , R. Bansal , S. R. Shin , Micromachines 2020, 11, 849. 32932680 10.3390/mi11090849 PMC7570325

[270] S. Maharjan , B. Cecen , Y. S. Zhang , Small Methods 2020, 4, 2000235. 33072861 10.1002/smtd.202000235 PMC7567338

[271] F. Sharifi , Su Su Htwe , M. Righi , H. Liu , A. Pietralunga , O. Yesil‐Celiktas , S. Maharjan , B. H. Cha , Su R Shin , M. R. Dokmeci , N. E. Vrana , A. M. Ghaemmaghami , A. Khademhosseini , Y. S. Zhang , Adv. Healthcare Mater. 2019, 8, 1801425. 10.1002/adhm.201801425 PMC6398437 30694616

[272] M. Ungemach , T. Doll , N. E. Vrana , Eur. J. Immunol. 2019, 49, 517. 30942902 10.1002/eji.201970045

[273] S. Yadavali , H. H. Jeong , D. Lee , D. Issadore , Nat. Commun. 2018, 9, 1222. 29581433 10.1038/s41467-018-03515-2 PMC5964316

[274] E. L. Schneider , J. Henise , R. Reid , G. W. Ashley , D. V. Santi , Bioconjug. Chem. 2016, 27, 1210. 26930186 10.1021/acs.bioconjchem.5b00690

[275] J. M. Lim , N. Bertrand , P. M. Valencia , M. Rhee , R. Langer , S. Jon , O. C. Farokhzad , R. Karnik , Nanomed.: Nanotechnol. Biol. Med. 2014, 10, 401. 10.1016/j.nano.2013.08.003 PMC3951970 23969105

[276] E. Kang , Y. Y. Choi , S. K. Chae , J. H. Moon , J. Y. Chang , S. H. Lee , Adv. Mater. 2012, 24, 4271. 22740066 10.1002/adma.201201232

[277] A. Díaz Lantada , W. Pfleging , H. Besser , M. Guttmann , M. Wissmann , K. Plewa , P. Smyrek , V. Piotter , J. García‐Ruíz , Polymers 2018, 10, 1238. 30961163 10.3390/polym10111238 PMC6401721

[278] A. E. Ongaro , D. Di Giuseppe , A. Kermanizadeh , A. Miguelez Crespo , A. Mencattini , L. Ghibelli , V. Mancini , K. L. Wlodarczyk , D. P. Hand , E. Martinelli , V. Stone , N. Howarth , V. La Carrubba , V. Pensabene , M. Kersaudy‐Kerhoas , Anal. Chem. 2020, 92, 6693. 32233401 10.1021/acs.analchem.0c00651

[279] P. S. Gromski , J. M. Granda , L. Cronin , Trends Chem. 2020, 2, 4.

[280] A. J. Mijalis , D. A. Thomas , M. D. Simon , A. Adamo , R. Beaumont , K. F. Jensen , B. L. Pentelute , Nat. Chem. Biol. 2017, 13, 464. 28244989 10.1038/nchembio.2318

[281] M. Panza , S. G. Pistorio , K. J. Stine , A. V. Demchenko , Chem. Rev. 2018, 118, 8105. 29953217 10.1021/acs.chemrev.8b00051 PMC6522228

[282] Y. J. Heo , D. Lee , J. Kang , K. Lee , W. K. Chung , Sci. Rep. 2017, 7, 11651. 28912565 10.1038/s41598-017-11534-0 PMC5599535

[283] H. J. Albers , R. Passier , A. van den Berg , A. D. van der Meer , Micromachines 2019, 10, 781. 31739604 10.3390/mi10110781 PMC6915557

[284] S. Mao , W. Li , Q. Zhang , W. Zhang , Q. Huang , J. M. Lin , TrAC, Trends Anal. Chem. 2018, 107, 43.

[285] L. R. Soenksen , T. Kassis , M. Noh , L. G. Griffith , D. L. Trumper , Lab Chip 2018, 18, 902. 29437172 10.1039/c7lc01223c PMC9011357

[286] D. Welch , J. B. Christen , Lab Chip 2014, 14, 1191. 24493132 10.1039/c3lc51205c

[287] S. M. Czerniecki , N. M. Cruz , J. L. Harder , R. Menon , J. Annis , E. A. Otto , R. E. Gulieva , L. V. Islas , Y. K. Kim , L. M. Tran , T. J. Martins , J. W. Pippin , H. Fu , M. Kretzler , S. J. Shankland , J. Himmelfarb , R. T. Moon , N. Paragas , B. S. Freedman , Cell Stem Cell 2018, 22, 929. 29779890 10.1016/j.stem.2018.04.022 PMC5984728

[288] R. Novak , M. Ingram , S. Marquez , D. Das , A. Delahanty , A. Herland , B. M. Maoz , S. S. F. Jeanty , M. R. Somayaji , M. Burt , E. Calamari , A. Chalkiadaki , A. Cho , Y. Choe , D. B. Chou , M. Cronce , S. Dauth , T. Divic , J. Fernandez‐Alcon , T. Ferrante , J. Ferrier , E. A. Fitzgerald , R. Fleming , S. Jalili‐Firoozinezhad , T. Grevesse , J. A. Goss , T. Hamkins‐Indik , O. Henry , C. Hinojosa , T. Huffstater , K. J. Jang , V. Kujala , L. Leng , R. Mannix , Y. Milton , J. Nawroth , B. A. Nestor , C. F. Ng , B. O'connor , T. E. Park , H. Sanchez , J. Sliz , A. Sontheimer‐Phelps , B. Swenor , G. Thompson , G. J. Touloumes , Z. Tranchemontagne , N. Wen , M. Yadid , A. Bahinski , G. A. Hamilton , D. Levner , O. Levy , A. Przekwas , R. Prantil‐Baun , K. K. Parker , D. E. Ingber , Nat. Biomed. Eng. 2020, 4, 407. 31988459 10.1038/s41551-019-0498-9 PMC8011576

[289] C. Probst , S. Schneider , P. Loskill , Curr. Opin. Biomed. Eng. 2018, 6, 33.

[290] A. Vasilevich , J. de Boer , Curr. Opin. Biomed. Eng. 2018, 6, 74.

